# Ventilation and filtration strategies to reduce respiratory infections in schools: a scoping review

**DOI:** 10.3389/phrs.2026.1608923

**Published:** 2026-07-16

**Authors:** Elisabetta Erriu, Jennifer Hasselgard-Rowe, Elsa Gaillard, Antoine Flahault

**Affiliations:** 1 Faculté de Médecine, Institut de Santé Globale, Université de Genève, Geneva, Switzerland; 2 Service de Sante de la Jeunesse, Geneva, Switzerland

**Keywords:** air filtration, indoor environment, mechanical ventilation, natural ventilation, respiratory infections

## Abstract

**Objective:**

This scoping review examines evidence on ventilation and filtration strategies in schools and their association with respiratory infection incidence and proxy indicators such as CO2, particulate levels, and absenteeism.

**Methods:**

Following PRISMA-ScR guidelines, we searched PubMed, Embase, and Web of Science (19 March 2025) for studies published between 2020 and 2025 in English, French, or Italian. Eligible designs included interventional, observational, and modelling studies, as well as reviews. Screening and data extraction were performed using Rayyan, and similar publications were verified with LiteRev.

**Results:**

Sixty-two studies met inclusion criteria. Mechanical ventilation, especially with high-efficiency filtration, was associated with lower CO2, fewer airborne particles, and reduced infection incidence or absenteeism. Natural ventilation showed variable effectiveness influenced by climate and window use. Hybrid systems and multilayered approaches provided the greatest reductions in estimated infection risk.

**Conclusion:**

Ventilation and filtration strategies reduce respiratory infection risk in schools. Mechanical and hybrid systems perform more reliably than natural ventilation. Further research should address feasibility, safety, sustainability, and implementation in low-resource settings.

## Introduction

Airborne transmission of respiratory pathogens has been well documented for decades, long before the COVID-19 pandemic renewed global attention to this mode of spread [1]. Schools represent a particularly relevant setting for studying airborne transmission, as students and staff spend prolonged periods indoors—typically 7–8 h per day [2]—and 12% of their time in classrooms with limited ventilation options [3]. These conditions can facilitate the accumulation of exhaled aerosols and increase the risk of respiratory infections such as COVID-19, influenza, and RSV [4, 5]. Moreover, children are particularly vulnerable to air pollutants because of their physiology [2]. Multiple studies conducted before and during the pandemic have shown that many schools do not meet recommended ventilation standards. For example, the SINPHONIE study conducted across 23 European countries between 2010 and 2012 found that the majority of schools had CO2 concentration higher than the WHO guidelines (>1000 ppm) and 86% of the schools had natural ventilation systems, 7% had mechanical ventilation and 7% natural assisted exhaustion [2, 6]. In 2005, in France, in 40% of schools, when classrooms were occupied, the CO2 levels were higher than 1700 ppm [2] and according to a current EMPA (Eidgenössische Materialprüfungs-und Forschungsanstalt) study, in Graubünden, Switzerland, in 2021, 60% of the classrooms had CO2 levels above 2000 ppm during occupancy, indicating insufficient air renewal [7].

Indoor air quality (IAQ) in Europe affects over 64 million students and 4.5 million teachers [8]. Therefore, managing indoor air conditions is a major focal point for the development of infectious diseases prevention strategies. Poor indoor air quality has been identified as closely related to increased concentrations of aerosols and CO2, which contribute to higher infection risk [9–12]. Poor indoor air quality also decreases cognitive functions and causes absenteeism due to illness, elements that represent a significant health and economic burden (depending on which air pollutant the cost can go up to 22 billion euros, and the socioeconomic cost in France is estimated to be around 19 billion euros for poor indoor air quality) [1, 2, 10, 13, 14]. In Italy, between 2022 and 2023, there was a 46% increase in school absenteeism due to illness, reflecting the unusually high circulation of respiratory viruses during the post-pandemic period. In France, high school students lost on average between 7% and 8.8% of their educational class time, largely due to illness-related absences and repeated disruptions linked to respiratory infection waves [2].

Different interventional, observational and mathematical modeling studies consistently identify ventilation and filtration strategies as effective interventions to reduce CO_2_, aerosol concentrations, and estimated infection risk. Mechanical ventilation, together with air filtration systems, is considered crucial to reduce airborne contamination in schools by some authors [9, 10, 13, 15, 16], while natural ventilation has been used in different settings especially because of its low economic cost and simplicity and has been found to be effective especially with other measures [15, 17, 18]. Finally, a growing body of evidence promotes a multi-layered strategy, combining ventilation and filtration measures, CO2 monitoring, mask use and occupancy control. Hence, the consensus is still not fully shared; there are still issues regarding acceptance, for example of mask use, and therefore there are a number of different elements to take into consideration.

This scoping review synthesizes findings from 62 empirical, modeling and review-based studies published between 2020 and 2025, focusing specifically on ventilation and filtration strategies applied in schools and their association with respiratory infection incidence and related proxy indicators (CO_2_, particulate matter, absenteeism). The goal is to identify effective and adaptable solutions that can help schools to maintain safe indoor environments. This review responds to an urgent need for evidence-informed strategies to safeguard the health of students and staff in educational institutions.

Ultimately, improving indoor air quality in school settings is not only a post-pandemic response, but a long-term investment in health and wellbeing for future generations [19–21]. As respiratory health challenges persist, resilient and efficient indoor air quality methods must remain a top priority in educational facility design and management [22–25]. The collective findings presented here offer a comprehensive roadmap toward that goal.

## Methods

### Protocol and reporting frameworks

This scoping review followed the *Preferred Reporting Items for Systematic Reviews and Meta-Analyses for Scoping Reviews* (PRISMA-ScR) guidelines. No protocol was registered in advance (e.g., OSF), which represents a methodological limitation; however, all steps of the review process are transparently reported below to ensure reproducibility.

### Conceptual focus: exposure and outcomes

Given the heterogeneity of the literature, we explicitly defined the exposure of interest as ventilation and air-filtration strategies implemented in school environments (mechanical ventilation, natural ventilation, hybrid systems, HVAC optimization, portable air cleaners) for respiratory infections (upper and lower respiratory tract infections). We did not include studies focusing primarily on chemical indoor pollutants unrelated to ventilation.

The outcomes of interest were:

1. Incidence of respiratory infections (COVID-19, influenza, RSV, acute upper/lower respiratory infections),
2. Proxy indicators of airborne exposure, including CO_2_ concentration, particulate matter levels, aerosol decay,
3. School absenteeism when explicitly linked to respiratory illness.

### Search strategy

Relevant publications were searched for through PubMed, Embase and Web of Science databases (Core Collection + Emerging Sources Citation Index + Science Citation Index Expanded). The inclusion of multiple WoS sub-databases explains the higher number of retrieved records compared with PubMed, particularly due to engineering and physics journals publishing modelling studies on ventilation. The systematic search was conducted on 19.03.2025 by the main author. The search combined controlled vocabulary and free-text terms related to ventilation strategies and respiratory infections. The full database-specific search strings are provided in Supplementary Material. A combination of the following words was used to find relevant studies in the existing literature:

“Indoor air,” “indoor air quality,” “air circulation” “air recirculation” “air filtration,” “ventilation,” “natural ventilation,” “mechanical ventilation,” “COVID-19,” “grippe,” “human flu,” “influenza*,” “RSV infection,” “school,” “school*,” “school building,” “university,” “educational building”.

### Inclusion and exclusion criteria

The included publications had to be written in English, French and Italian and published within the study period of 2020 and 2025. This period was selected because the COVID-19 pandemic generated a substantial increase in empirical and modelling studies on ventilation in schools, making it the most relevant timeframe for current policy discussions. Language restrictions were applied based on the research team’s linguistic proficiency to ensure accurate interpretation of methodological details. Although ventilation research predates COVID-19, pre-2020 studies rarely focused specifically on school settings or respiratory infection outcomes. The period 2020–2025 was therefore selected because it corresponds to the highest volume of relevant, school-based empirical and modelling evidence, making it the most appropriate timeframe for this review. The scientific papers had to be original research articles, reviews, modelling studies, interventional and observational studies, or clinical studies and trials, RCT and guidelines. The settings included schools, universities or educational buildings. The outcomes included were respiratory infections or proxy indicators (CO_2_, aerosol concentration, absenteeism).

The exclusion criteria were studies not conducted in educational settings, studies focusing solely on chemical pollutants unrelated to ventilation, and commentaries, editorials, and non-scientific reports.

The original research was conducted on 19.03.2025 and it resulted in: 28 results for PubMed, 39 results for Embase and 266 results for Web of Science. In total there were 333 articles, of which seven pertinent reviews and 59 duplicates.

### Data extraction

The identified dataset was extracted using Rayyan web-tool and the duplicates were removed performed using Rayyan’s automated detection tool, followed by manual verification.

First, the reviews were searched by screening titles and abstracts. Seven reviews were found on the relationship between ventilation strategies-indoor air quality improvement and COVID-19.

The remaining publications were imported into Rayyan web-tool where the screening based on titles and/or abstracts was completed by the main author; to mitigate this, ambiguous cases were discussed with a second reviewer. 198 publications were excluded.

In the second stage, the full text of selected articles was read, and 55 articles and seven reviews were considered for this scoping review. A total of 62 studies met the inclusion criteria. To ensure completeness, we used “LiteRev”^1^, a machine-learning–based tool developed at the University of Geneva, which identifies publications similar to a predefined set of included articles. “LiteRev” did not identify additional eligible studies but confirmed the consistency of the final selection.

**FIGURE 1:**
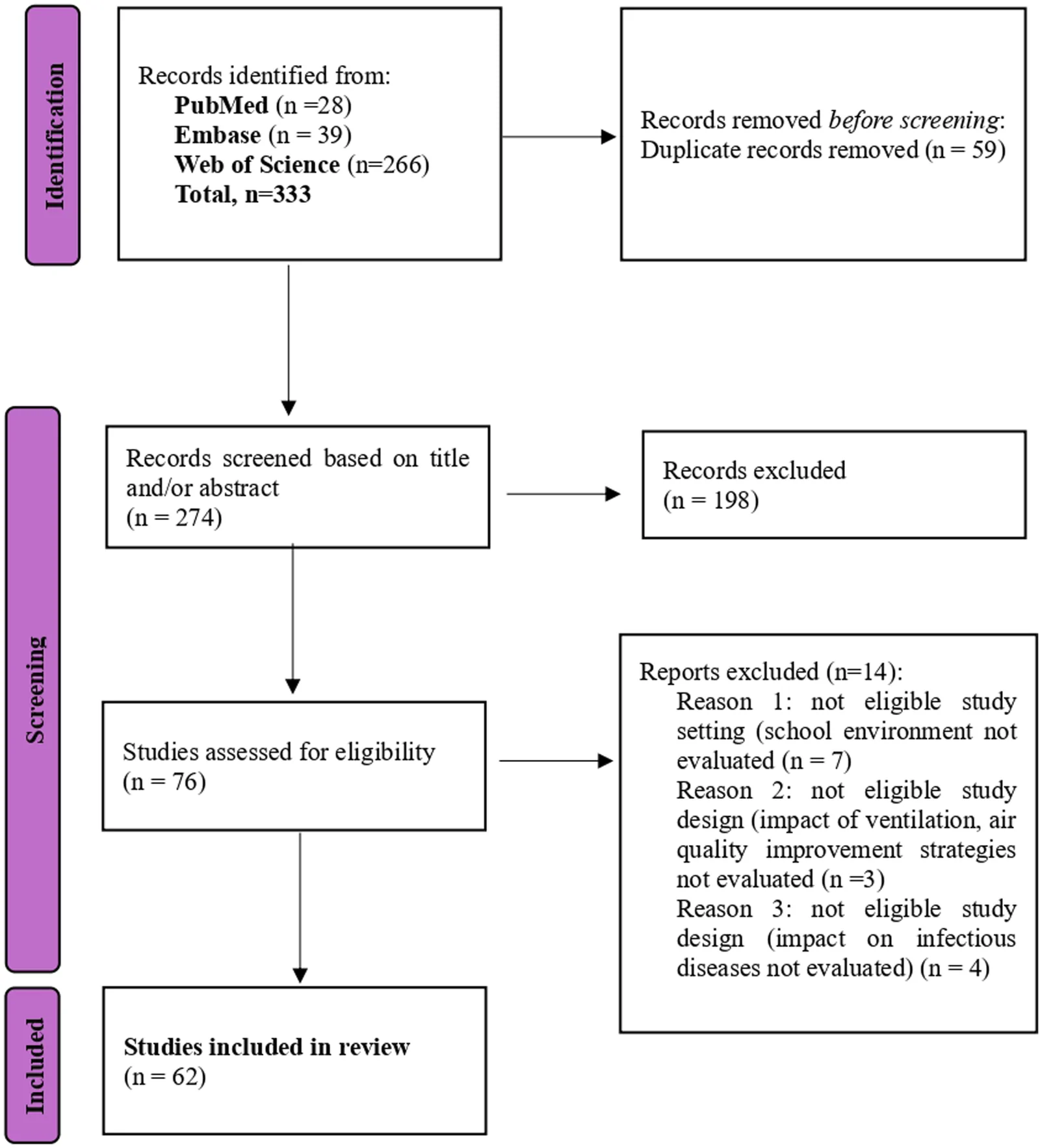
PRISMA- ScR 2020 flow diagram.

A flowchart describing the process of study selection is shown below in Figure 1.

Relevant data were later extracted into a structured Word table, which included: title, author(s) names, date of publication, methods, aim, study period of investigation, type of indoor setting, city, main results, type of ventilation used and main limitations of the study (“Supplementary Appendix 3” in “Supplementary Material”). Extraction was performed by one reviewer (EE) and cross-checked by a second reviewer (AF).

## Results

As described in the PRISMA-ScR flow diagram (Figure 1), the research carried out on 19.03.2025 identified a total of 333 records (PubMed n = 28; Embase n = 39; Web of Science n = 266) among which 59 duplicates were removed. After an initial screening of 274 titles and abstracts and then 76 full-text screening, a total of 212 publications were excluded. We identified 62 publications in total that met our inclusion criteria. Of these, one was published in 2025 [1], 11 in 2024 [4, 5, 9–13, 17, 18, 35], 20 in 2023 [15, 16, 28, 29, 31, 32, 34, 36, 42, 46, 48–51, 55, 56, 58, 64, 67, 68], ten in 2022 [22–24, 26, 30, 33, 37, 41, 54, 57, 63, 65, 66], 19 in 2021 [19–21, 27, 38–40, 43–45, 47, 52, 53, 59, 60, 62], one in 2020 [25].

Of the 55 articles included, without considering the seven reviews: the study locations of the articles were as follows: 32 in Europe [1, 4, 10, 12, 15, 16, 20, 21, 26, 29–33, 35, 37, 38, 42–44, 46, 47, 52, 53, 55–60, 64, 65]; 11 in the USA [9, 13, 22, 25, 27, 28, 39–41, 61, 67]; 2 in Latin America [5, 54]; six in Asia [11, 45, 49–51, 68]. Four articles did not provide a specific location as they were mathematical modeling studies [23, 62, 63, 67].

In terms of study design, five publications were an “Intervention study” (“Supplementary Table 1” in “Supplementary Appendix 1” in “Supplementary Material”) [9, 15, 26, 36, 44], 17 were an “Observational study” (“Supplementary Table 2” in “Supplementary Appendix 1” in “Supplementary Material”) [12, 13, 28, 32, 33, 35, 37, 38, 42, 43, 47, 53, 57–61], 17 were an “Observational study and mathematical modeling study” (“Supplementary Table 3” in “Supplementary Appendix 1” in “Supplementary Material”) [1, 5, 11, 16, 20–22, 27, 39, 45, 52, 54–56, 64, 65, 68], 16 were a “Mathematical modeling study” (“Supplementary Table 4” in “Supplementary Appendix 1” in “Supplementary Material”) [4, 10, 23, 25, 29–31, 40, 41, 46, 49–51, 62, 63, 67] and seven were Reviews (“Supplementary Table 5” in “Supplementary Appendix 1” in “Supplementary Material”) [17–19, 24, 34, 48, 66] (“Supplementary Tables 1–5” in “Supplementary Material in Supplementary Appendix 1”).

Interventional settings included implementing additional ventilation strategies, for example adding mechanical ventilation systems (air cleaners, filters) to classrooms that were only naturally ventilated [15, 26, 36]; combining natural ventilation with mechanical ventilation strategies [36]; or comparing HVAC (heating, ventilation and air conditioning) systems to fan coil units [9].

Observational settings included ventilation measures collected on-site (indoor air quality measurement, CO2 concentration) [1, 5, 11, 12, 20–22, 32, 33, 35, 42, 43, 47, 52–60, 64, 65, 68]; collection of environmental, epidemiological and molecular data [16]; natural and mechanical ventilation measures [27, 37, 38]; CO2 decay by cross and single-sided ventilation with ratio of windows opening [45]; collection of COVID-19 cases [13, 61]; students’ attendance records to evaluate absenteeism [28]; and surveys of preventive measures [39].

Mathematical settings included scenario-based analysis and infection risk modeling [4, 29, 30, 40, 62, 67]; using Wells-Riley equations [10, 23, 25, 31, 41, 49, 50]; aerosol transmission models [63]; Monte-Carlo simulation models [46]; Eulerian-Lagrange methods [51].

It is possible to divide the results from the 62 publications into different categories, according to the effectiveness of measures/strategies to reduce airborne transmission of infectious diseases in schools:

1. Mechanical ventilation (with or without filtration, and double flow ventilation systems): we found that mechanical ventilation (with or without filters) is significantly associated with lower incidence of respiratory infections and contributes to a better control of CO2 levels and particle removal [20, 22, 27, 28, 32, 33]. In addition, it is also effective with other interventions, such as filtration and mask use; and more efficient than natural ventilation alone [26, 31].
2. Air filtration and purifiers (HEPA (High-efficiency particulate air), PECO (Photoelectrochemical oxidation), MERV (Minimum efficiency reporting values): highly effective in reducing infected particle concentration and viral load in indoor school air. It is also beneficial when natural ventilation is insufficient and cannot be guaranteed at a safe level, but it does not replace the need for fresh air [16, 36, 37].
3. Hybrid ventilation (natural and mechanical): it showed lower CO2 levels and infection risk especially in seasonal-sensitive climates. It is more effective than natural ventilation alone. In Vouriot et al. [21] hybrid ventilation cut transmission risk of COVID-19 by half between winter and summer, and Pollozhani et al. [4], Albertin et al. [46], Zivelonghi et al. [52] and Alonso et al. [47] show reduced estimated infection risk with hybrid ventilation and mask use.
4. Natural ventilation (windows and doors open): it is partially effective, depending on window opening ratio, orientation of the classroom, occupancy, activity level and seasonality. It does not guarantee a low level of viral particles. Park et al. [45] and Di Gilio et al. [53] found that natural ventilation only was insufficient to reduce the COVID-19 infection probability, and the risk was reduced by four times with mask usage.
5. Supplementary and mixed measures (HVAC optimization, mask, microphone use, occupancy control): these measures are essential modifiers which improve the performance of the ventilation strategy. Mask use added to natural ventilation often results in an infection risk below 1% [4, 45, 52].

Table 1 summarizes the main ventilation strategies, typical outcomes, nature of the evidence and representative studies. Mechanical ventilation and filtration strategies showed the most consistent benefits across study designs, while natural ventilation demonstrated context-dependent effectiveness.

**TABLE 1:** Summary of ventilation and filtration strategies, typical outcomes, and representative studies.

Ventilation strategy	Typical result	Nature of evidence	Citation example
Mechanical ventilation	Consistent association with lower CO_2_, reduced aerosol concentration, and lower estimated respiratory infection risk. Some studies also report reduced absenteeism	Evidence largely consistent across observational, interventional, and modelling studies. Few contradictory findings	[9, 10, 20, 22, 28, 30, 31, 36, 37, 45, 47, 50, 56] [26]
Air filtration (HEPA, MERV, PECO)	Reduction in airborne particle concentration and viral load; may reduce infection incidence or absenteeism when properly sized and maintained	Mostly consistent evidence; some concerns about noise, risk compensation, and limited effect on CO_2_	[1, 9, 12, 13, 15, 16, 25, 27, 43, 46, 48, 57, 59, 60, 67]
Hybrid ventilation (natural + mechanical)	Balance between efficiency and feasibility. Improved air renewal compared with natural ventilation alone; more stable performance across seasons; associated with lower CO_2_ and reduced infection risk	Evidence consistent across modelling and empirical studies; effectiveness depends on climate and building characteristics	[4, 21, 26, 34, 44, 55, 58, 63]
Natural ventilation	Highly variable effectiveness; may reduce CO_2_ and aerosol levels when windows are sufficiently opened; performance strongly influenced by climate, occupancy, and window configuration	Evidence mixed. Several studies show insufficient air renewal or limited infection risk reduction when used alone. Effectiveness improves with masks, cross-ventilation, or reduced occupancy	[5, 12, 23, 32–35, 38–40, 51, 52, 62, 64–66]
Supplementary strategies (mask use, occupancy control, CO_2_ monitoring, microphone use, HVAC optimization)	Enhance the effectiveness of all ventilation strategies; mask use consistently associated with large reductions in estimated infection probability	Strong modelling evidence; empirical evidence more limited but generally supportive	[4, 11, 40, 41, 49, 53, 54, 59, 61, 62, 68]

Definition of categories: Outcomes were categorized based on whether studies reported (1) CO_2_ reduction, (2) aerosol/particle reduction, (3) estimated infection probability, or (4) absenteeism.

Interpretation of “typical outcome”: Terms such as “associated with lower CO_2_” or “may reduce infection risk” reflect consistent patterns across studies, not causal certainty.

Contradictory findings: Natural ventilation showed the greatest variability, with several studies reporting insufficient air renewal in winter or in densely occupied classrooms.

## Discussion

The aim of the present scoping review is to evaluate ventilation strategies and air-filtration interventions in school settings and their association with respiratory infection incidence and related proxy indicators (CO_2_, aerosol concentration, absenteeism). As highlighted by the results illustrated in the previous section, there is a consensus that indoor air quality improvement is associated with a better infectious disease epidemiological outcome. Findings were generally consistent across interventional, observational, and modelling studies, although modelling studies tended to report larger estimated effects due to idealized assumptions.

Even though some publications have emphasized the relationship between indoor air quality and COVID-19, few have specifically focused on other infectious diseases and on evaluating this relationship in school settings. Yet, the research is growing thanks to the important knowledge brought by the pandemic period.

In the 62 publications selected for this scoping review, CO2 levels were measured. This highlights the importance of CO2 concentration as a proxy for respiratory infectious diseases; however, it is argued that, for instance, COVID-19 virus is transmitted via small droplets of fluid. Therefore, the movement and dispersion of COVID-19 are more complex and highly dependent on droplet size compared to CO2 [54]. To decrease the CO2 concentration in classrooms, different recommendations have been proposed by the World Health Organization and the US Centers for Disease Control and Prevention [18]: indoor CO2 levels should not exceed 1000 ppm on average [34]. This can be implemented using different ventilation strategies studied in the literature: mechanical ventilation, natural ventilation or mixed measures.

### Mechanical ventilation: consistent benefits but variable feasibility

Our literature search confirms previous findings that mechanical ventilation is crucial to reduce airborne contamination in schools [9, 10, 34]. In fact, the risk of COVID-19 and other respiratory infections outbreaks in school should not be underestimated due to the fact that many children are not vaccinated and lack other preventive measures [11]. Busato and Cavallini [31] showed that mechanical ventilation, with or without a mask, reduced the infection risk of COVID-19 by three times or more, in different Italian classrooms compared to opening windows, and could even outperform the effect of solely wearing a mask. The retrospective cohort study of Buonanno et al. [26] reveals how mechanical ventilation strategies protected students from airborne exposure leading to infection of COVID-19 in more than 1400 school in Italy. For classrooms equipped with mechanical ventilation systems, the relative risk of infection of students decreased at least by 74% compared with a classroom with only natural ventilation, reaching values of at least 80% for ventilation rates >10 L s^-1^ student^-1^. Moreover, only 31 students became ill in classes with mechanical ventilation compared to 3090 in classes without, during the period of September 2021 and January 2022 [26]. In addition, mechanically ventilated classrooms seemed to have lower CO2 levels [34] and increased ventilation rates, which correlates with reduced infection probability in schools; however, they may need to be balanced with energy use [28]. At this purpose, double flow ventilation systems are recommended since they allow energy savings through heat recovery systems [2]. Mechanically ventilated classrooms also had lower levels of PM2.5 and the observational study of Deng et al. [28] found that every 1L/s increase per person in ventilation rates through mechanical ventilation corresponds to a 5·59 decrease in absent days per year (which translates into a 0·15% increase in the annual daily attendance rate). This clearly supports the recommendation that schools should be equipped with mechanical ventilation.

Overall, mechanical ventilation was associated with: lower CO_2_ concentrations [28, 34], reduced aerosol or particle levels [20, 22, 27], lower estimated infection probabilities [26, 31], and, in some studies, reduced absenteeism [28]. These findings align with long-standing evidence that adequate air exchange reduces airborne pathogen accumulation [9, 10]. Mechanical systems also provide stable performance across seasons, unlike natural ventilation, which is highly weather-dependent [45, 52]. However, feasibility varies. Mechanical systems require: installation and maintenance cost [2], trained personnel [34], energy consumption considerations [28], and infrastructure that many older school buildings lack [2]. In low-resource settings, these constraints may limit implementation, highlighting the need for low-cost ventilation alternatives. Low-cost strategies for LMICs could include structured window-opening protocols, low-cost CO_2_ monitors, locally manufactured HEPA units, simple architectural modifications (roof vents, cross-ventilation corridors) and improved classroom layout to enhance airflow [23, 25].

### Air filtration: effective complement, not a substitute

A significant number of publications found air filtration-purification systems, particularly HEPA and MERV-13, often used in combination with mechanical ventilation filters, showed strong reductions in aerosol concentrations [16, 37, 63] and, in some cases, respiratory infection incidence or absenteeism [13, 39]. Filtration was especially useful when natural ventilation was insufficient (cold climates, polluted outdoor air, security concerns) [1, 36], to be effective in reducing the transmission risk of infectious diseases.

A recent pre-print publication of 2025 (RCT, not included in our selection of published papers in this review) showed that air purifiers reduced pollution in 95 Italian classrooms by 28% and students’ absenteeism by 11% [69]. These devices significantly reduce airborne particle concentration regardless of the season or ventilation behavior [1] with the aim of controlling respiratory symptoms and infection rates [13, 15, 16], but their efficiency also depends on the type of air purifier and filter class [34]. Three types of air filtration devices were examined in the 62 publications: HEPA, MERV and PECO filters.

The observational and mathematical modeling study of Banholzer et al. [16] examined the effect of HEPA filters on school absenteeism in two Swiss secondary school classes during 7 weeks in winter: there were 22 absences related to respiratory infections without air cleaners and 13 with air cleaners. The researchers also estimated the number of respiratory infections in schools without air cleaners (36) as well as in cases where air cleaners had been installed during the study period (19) [16]. Granzin et al. [37] further showed that air purifiers with HEPA filters reduced the number of inhaled viral particles by a factor of 2·65, from November 2020 to May 2021, and Villiers et al. [63] found that the use of HEPA filters combined with students using face masks could reduce the mean aerosol concentration by 90%. In Gettings et al. [39], which evaluated the impact of preventive strategies against COVID-19 in 169 schools in Georgia (USA), the incidence of the disease was 39% lower in schools with improved ventilation, in particular 48% lower with HEPA (with and without purification UVGI) [39] versus 27% and 29% reduction respectively in Shen et al. [23] and Azimi et al. [25]. However, Falkenberg et al. [15] indicate that the use of HEPA filters might lead to a reduction in preventive behaviors because of the sense of security that they procure. Nevertheless, other studies conducted to examine the effectiveness of air filters to reduce indoor aerosol transmission agree on the positive effect of HEPA filters. In fact, compared to HVAC only systems which are designed to recirculate the air from one place to another, HEPA filters are relatively affordable and improve aerosol clearance [70].

The conclusion from the publications related to air filtration systems was that the use of filters with higher MERV ratings ensures an infection risk below 1% [40]. Indeed, Zand et al. [13] show how classrooms designed with MERV-13 filters had a lower COVID-19 incidence and positive PCR tests compared to MERV-11 [13], while Azimi et al. [25] revealed a 45% transmission risk for MERV-8 and 32% for MERV-13 [25]. Finally, only one study evaluated the efficiency of PECO air purifiers, and it showed a 99·98% removal of COVID-19 in an air chamber [36].

Despite all of the above, it is important to consider that HEPA filters do not remove gaseous pollutants and it is difficult to measure the exact effect of air filtration in classrooms because of the existence of confounding factors, such as the presence of other particles not emitted by humans [34], and there is a limitation to maintaining good indoor air quality when the outdoor air is very polluted [69]. Also, filtration does not provide fresh air [34], it does not remove CO_2_ [34], and its effectiveness depends on proper sizing, placement, and maintenance [16, 37]. Some studies also noted concerns about noise levels [15], which may affect classroom comfort, and the potential for risk compensation, where occupants reduce other preventive behaviours when filters are present [15].

### Natural ventilation: context-dependent and seasonally constrained

Natural ventilation (having windows and doors open) has been used in different studies [5, 54–57], but its role is controversial. Some publications show that it is not the most effective way to reduce transmission risk of infectious diseases in schools [49, 50, 58]. In France, according to a study by Cerema, the common practice of windows ventilation is to open the window for 5 min every 2 h. The minimum aeration time for good indoor air quality is 5 min every hour and 5 min for every break. This would add an extra cost of between 2 and 4 euros per student per year [2]. Windows ventilation is therefore considered more expensive and less efficient, considering that ventilation with heat recovery (double flow systems) would bring an economic gain between 6 and 7 euros per student per year and a social gain of better health, less absenteeism and better productivity [2]. The effectiveness of natural ventilation is often related to climate-sensitive regions and windows use, where in cold climates or densely populated areas its role ends up being insufficient, and many real-world case studies underscore the need for more reliable or combined alternatives [45, 52, 53]. The role of natural ventilation can be improved with the implementation of other measures, such as students wearing face masks or reducing the number of students in a class. Zivelonghi et al. [52] showed that natural ventilation could be more efficient for preventing transmission when combined with microphone use (20% risk reduction due to the reduced need to project the voice, which causes more droplets to spread), mask use (almost 40% risk reduction) and reduced children’s density (50% risk reduction). Moreover, Park et al. [45] proposed the use of cross ventilation (two or more openings on opposite or adjacent walls) compared to single ventilation (openings on the same wall) [45].

Overall, natural ventilation showed high variability in effectiveness [5, 12, 23, 49, 50]. Its performance depended on: window opening ratio [45], classroom orientation [53], occupancy density [52], outdoor temperature [45], and air pollution levels [54].

In cold climates, windows are often kept closed, limiting air renewal [45]. In hot climates, windows may be open but outdoor pollution or noise may reduce feasibility [54]. Seasonal constraints were highlighted in several studies, confirming that natural ventilation alone is unlikely to maintain adequate air quality year-round [52, 53].

Nevertheless, natural ventilation can be enhanced through cross-ventilation [45], scheduled airing [2], reduced occupancy [11], and mask use [45, 52].

### Hybrid ventilation: balancing performance and feasibility

Finally, a growing body of evidence promotes a multi-layered strategy to prevent the rise of infections, combining ventilation and filtration measures, CO2 monitoring, mask use and occupancy control. For example, Pollozhani et al. [4] considered adding mask use to mechanical and hybrid ventilation strategy, and Yen at al [11]. studied the reduction of the number of occupants in the room and the duration of their stay, finding both a strong reduction in transmission risk of COVID-19. Yet, even if some proposals such as using a microphone or implementing social distancing measures have been tested, the use of a mask, in combination with ventilation strategies, has been recognized as the most efficient supplementary strategy. In Harrington et al. [64], the probability of infection transmission was reduced from 75%–100% to 18%–43% over an 8-h school day with mask use. According to Gettings et al. [39] there was also a difference in incidence of COVID-19 if the teacher or students wore the masks: a 37% lower incidence rate when the teachers used face masks compared to a 21% rate if students wore a mask. Overall, hybrid systems combining natural and mechanical ventilation provided: improved air renewal [21], lower CO_2_ levels [21, 45], and reduced infection risk across seasons [4, 46, 47, 52].

These systems may offer a cost-effective compromise, particularly in temperate climates where natural ventilation can be used part of the year [21]. They also allow schools to reduce energy consumption by alternating between modes [28].

However, while significant progress has been made to understand the role of improving indoor air quality in infectious disease prevention in schools, disparities persist in implementation. In fact, even though in the 62 publications selected for this scoping review, mechanical ventilation with and without filters was found to be a very efficient strategy [26, 30, 40], it is important to consider that there are many lower-resourced schools around the world which often lack mechanical systems and real-time monitoring capacities. So, it is necessary to take these differences into consideration and, as far as possible, opt for low-cost, effective alternatives [33, 37, 44, 52, 53, 57, 60, 62]. This is the reason why some researchers have proposed hybrid ventilation as a balance between the efficiency and feasibility of reducing transmission risk [4, 35, 46].

### Supplementary measures: masks, occupancy, and behavioural strategies

Across modelling and empirical studies, mask use consistently produced large reductions in infection probability [4, 11, 45, 52, 64], often exceeding the effect of ventilation alone. Occupancy reduction [11], microphone use (reducing voice projection) [52], and CO_2_ monitoring [4] also improved outcomes.

These findings support a multi-layered approach, where ventilation is combined with behavioural and organizational measures [4, 11, 39].

### Safety, noise, and sustainability considerations

Few studies explicitly addressed safety concerns, such as: noise from portable air cleaners [15], thermal comfort when windows are opened [2], or the carbon footprint of mechanical systems [28].

These factors are important for policymakers and may influence acceptability among teachers and students [15]. Future research should evaluate: long-term energy-health trade-offs [28], noise mitigation strategies [15], and the environmental impact of different ventilation technologies [2].

### Limitations

Nonetheless, despite the fact that schools are well known to be settings for the spread of airborne infectious diseases, there are different limitations to the comparison between ventilation strategies in this scoping review.

First, it is difficult to compare the results of interventional, observational and mathematical modeling studies because of their different natures. Moreover, in the 62 publications analyzed, there was no general scheme for analyzing ventilation methods and risk transmission reduction, and the results were only qualitatively analyzed. For instance, for the interventional studies, mechanical ventilation was compared to natural ventilation strategies [26], HVAC systems were compared to fan coil units [9], and HEPA filters to natural ventilation [15, 36] and a combination of measures [44]. The 17 observational studies conducted indoor air quality measurements for different time periods, one study collected molecular data (bioaerosol and saliva samples) [16], only four studies examined COVID-19 cases [13, 15, 16, 61], two studies collected students attendance records (absenteeism) [16, 28], and one conducted a survey on preventive measures [39], and another on students’ activity levels [68]. Mathematical models mainly used the Wells-Riley equation, but others used the Monte Carlo [46] and Eulerian-Lagrange methods [51]. The Wells–Riley model is based on a different assumption that represents a limitation for the studies: indoor air and aerosols are well mixed and the steady state (which represents a constant concentration of quanta overt time, which may not be held in larger rooms or those with irregular shapes and varies with ventilation rates) [20, 45]. It also requires measurement of outdoor air supply rates [64].

Second, school settings vary significantly from one article to another, which might create some measurements’ bias: not only settings of different age groups (kindergarten, primary school, secondary school, university), but also different settings around the world (Europe, USA, Latin America, Asia) with specific and unreproducible characteristics (climate, temperature, humidity, building size and features). In fact, children of different ages are exposed to significantly different environmental conditions (such as physical environments and activities) [12] and differences in height and body weight between children and adults modify the CO2 concentration [11]. Most included studies were conducted in high-income countries [1, 4, 9–13, 15–18]. Evidence from LMICs is scarce, despite the fact that many schools in these settings rely exclusively on natural ventilation and have limited resources for mechanical systems [23, 25].

Third, in many articles it was not revealed whether the school setting had preventive measures in addition to the ventilation strategies that were implemented, and in some schools mask use and vaccines were already applied. This could influence the accuracy of the results and could be a confounding factor. For instance, in [12, 13, 15, 26, 29, 33, 39, 56, 61, 64], there were a number of possible bias, such as the use of masks during lessons (but not for preschool children [12, 59] and only for 30% of students and all the staff [13]), infection control strategies (frequent hand hygiene and cleaning of surfaces [12]) and maximum physical distance (1·5 m distance at least) [12].

Finally, the lack of published experimental trials is a relevant limitation of this scoping review and further research should be conducted. Additionally, this scoping review does not contain randomized clinical trials (except for one 2025 pre-print study mentioned in the discussion section [69]), as they are unfortunately uncommon in this domain. Therefore, we need more experimental research to assess the relationship between improved indoor air quality through ventilation strategies and the reduction of infectious diseases in schools.

Taken together, the evidence suggests that ventilation and filtration strategies may reduce airborne exposure and respiratory infection risk in schools [9–13, 15–18]. Mechanical and hybrid systems offer the most reliable performance [21, 26, 28, 52], while natural ventilation remains highly context-dependent [45, 52, 53]. Multi-layered approaches combining ventilation, filtration, and behavioural measures appear most effective [4, 11, 39, 52].

However, conclusions must remain cautious due to heterogeneity in study designs, outcomes, and modelling assumptions [1, 4, 10, 23].

### Conclusions

This scoping review highlights a growing body of evidence supporting the role of improved indoor air quality, ventilation and air-filtration interventions, in reducing respiratory infection incidence in school settings. Across diverse study designs, the findings suggest that improving air renewal through mechanical, hybrid, or optimized natural ventilation may contribute to reducing airborne exposure and respiratory illness risk among students and staff.

Mechanical ventilation, with and without filtration systems, or hybrid ventilation appears consistently more effective than natural ventilation alone in lowering infection risk, absenteeism and improving indoor air quality across empirical and modelling studies. Hybrid systems offered a balance between performance and feasibility, while natural ventilation demonstrated highly variable effectiveness depending on climatic, structural, and behavioural factors. Multi-layered approaches combining ventilation, filtration, masking, occupancy control, and CO_2_ monitoring appeared to provide the greatest overall protection.

Despite the promising findings, the outcome measures and heterogeneity of the study designs with limited real-world interventions, scarce data from low- and middle-income countries, few evaluations of long-term sustainability, noise, safety, or energy-health trade-offs, as well as different school environments, limit the generalizability of the results. There is a strong need for clear goals, up-to-date guidelines for indoor air quality in schools, transdisciplinary coordination and further scientific data to increase prevention and investments by governments. The main goal is to reduce the burden of respiratory infectious diseases in schools by improving indoor air quality in schools with solutions that can balance energy resources and good indoor air quality. The evidence in the literature analyzed here supports prioritizing indoor air quality improvements as part of a comprehensive prevention strategy. Overall, the available evidence suggests that ventilation and filtration strategies represent essential components of healthier and more resilient school environments. Future work should also address safety, noise levels, and the carbon footprint of ventilation systems, as well as absenteeism, which remains an important but underreported outcome. Strengthening these measures—while ensuring feasibility, equity, and sustainability—may contribute to reducing the burden of respiratory infection and promoting healthier learning environments for students and staff.

## Footnotes

1
https://literev.unige.ch/
