# Supplementary material for: Ventilation and filtration strategies to reduce respiratory infections in schools: a scoping review
Source: Public Health Rev. 2026 Jul 16;47:1608923. doi: 10.3389/phrs.2026.1608923 (PMC13454833; doi:10.3389/phrs.2026.1608923)
Supplement: Supplementary file 1 [file Supplementaryfile1.docx]

**Supplementary Material : Appendix 1**

**Table 1 : INTERVENTION STUDY:**

| **Title** | **Study type** | **Setting** | **Methods** | **Main results** | **Ventilation type** |
| --- | --- | --- | --- | --- | --- |
| Increasing ventilation reduces SARS-CoV-2 airborne transmission in schools: a retrospective cohort study in Italy’s Marche region | Intervention study  Retrospective cohort study | 1419 schools (10441 classrooms) in Marche region (Italy) | 56 schools (316 classrooms) equipped with mechanical ventilation | Classrooms with mechanical ventilation: 31 infected students. With natural ventilation: 3090 infected students. Mechanical ventilation systems are efficient to protect students from COVID-19 transmission | Natural and mechanical ventilation |
| A Room HVAC Influences on the Removal of Airborne Particulate Matter: Implications for School Reopening during the COVID-19 Pandemic | Intervention study with observational elements | 5 classrooms of Clemson University (USA) | Measurements and analytical calculations: experiments in 5 classrooms with different HVAC configurations (ventilation type, airflow rates, and filtration on IAQ) | Classrooms with central HVAC system responded better and faster for indoor air contamination compared to the ones with only fan coil units.  Increasing the ventilation rate without improving filtration is inefficient. Air filters are needed | Mechanical ventilation: HVAC system (heating, ventilation and air conditioning) |
| Effect of portable HEPA filters on COVID-19 period prevalence: an observational quasi-interventional study in German kindergartens | Quasi-interventional and observational study | 32 (10 intervention, 22 control) kindergartens in Rhineland  Palatinate (Germany) | Intervention group using HEPA (high efficiency  particulate air) filters and the control group not | Control group: 186 per 1,000 children. Intervention group: 372 per 1,000 children.  HEPA filters can significantly reduce the  viral concentration in the classroom air, but not for COVID-19 prevalence in the selected kindergartens in Germany | Air cleaners and HEPA filters |
| Efficient trapping and destruction of SARS-CoV-2 using PECO-assisted Molekule air purifiers in the laboratory and real-world settings | Intervention study | Test chamber | Laboratory experiment, University of Nebraska Medical Center (UNMC), USA | In 2 min, Molekule Air Mini+ removes 99.98 % of COVID-19 in an air chamber.  In 1 h, UV-A-stimulated PECO destructs over 99 % COVID-19 on filtration media.  Molekule Air-Pro air purifier removes COVID-19 from the air | Air filtration: PECO-assisted Molekule air purifiers |
| Implementation of a Ventilation Protocol for SARS-CoV-2 in a Higher Educational Centre | Intervention study | University center in Valladolid (Spain) | Experimental study in a typical classroom | Combining natural ventilation, air conditioning (with 50% of outdoor air) and filtering device was the best strategy at removing particles and aerosols fast | Natural, mechanical ventilation, air purifiers |

**Table 2: OBSERVATIONAL STUDY:**

| **Title** | **Study type** | **Setting** | **Methods** | **Main results** | **Ventilation type** |
| --- | --- | --- | --- | --- | --- |
| The Impact of Large Mobile Air Purifiers on Aerosol Concentration in Classrooms and the Reduction of Airborne Transmission of SARS-CoV-2 | Observational study | 1 primary school of 3 classrooms in Germany | Indoor air quality measurements and aerosol generator measurements: number of particles, particle size distribution, and the CO_2_ concentration | Room size, duration of stay, and activity of the people in the  Room: major influence on the inhaled dose of virus-laden particles.  The use of air purifier: positive effect in reducing virus-laden aerosols  when people are in small rooms for long periods.  Window ventilation reduces respiratory particle concentration and improves air  quality in terms of humidity and CO2, but it is not reliable  to reduce the concentration of virus-laden particles | Air purifiers (HEPA filters). Natural ventilation |
| Assessment of CO_2_ and aerosol (PM_2.5_, PM_10_, UFP) concentrations during the reopening of schools in the COVID-19 pandemic: The case of a metropolitan area in Central-Southern Spain | Observational study | 7 schools, 19 classrooms: 6 pre-schools, 7 primary and 6 secondary schools in Ciudad Real (Central-Southern Spain) | Indoor air quality monitoring and survey | Only cross ventilation does not seem to be efficient for providing adequate ventilation rates.  5 classrooms (26%) had CO2 concentration >700 ppm.  Better ventilation in pre-schools and secondary classrooms showed the highest peak and average CO2 concentrations. For PM2.5, PM10 and UFP: 2-fold greater in preschools | Cross natural ventilation (all windows and doors open). Only 1 classroom had mechanical ventilation |
| Indoor Air Quality in Naturally Ventilated Classrooms. Lessons Learned from a Case Study in a COVID-19 Scenario | Observational study | Nursery and primary public school in Valladolid (Castilla y León, Spain | Air quality and hygrothermal comfort conditions before and after the pandemic | Mixing model (ensures the removal of pollutants) is guaranteed by continuous cross-ventilation | Ventilation scenarios |
| Effects of the COVID-19 Pandemic on Indoor Air Quality and Thermal Comfort of Primary Schools in Winter in a Mediterranean Climate | Observational study | 1 elementary school (2 classrooms) in Sevilla (Spain) | Indoor air quality measurements before and after the COVID-19 pandemic | Decrease of 300 ppm in CO2 weekly average values during the  pandemic, when hybrid ventilation is used, and a decrease of 400 ppm when natural ventilation is added | Mechanical and natural ventilation |
| Testing mobile air purifiers in a school classroom: Reducing the airborne transmission risk for SARS-CoV-2 | Observational study | 1 high school (2 classrooms) in Germany | IAQ measurements with natural ventilation (door and up to 5 windows open- time interval venting) and with 3-4 air purifiers (HEPA filters) | Air purifiers are an effective measure to reduce the transmission risk of COVID-19:  With all windows and door closed within the intervals between venting by opening windows and door, the aerosol concentration was reduced by more than 90% in less than 30 min with the air purifier | Air purifiers (HEPA filters) and natural ventilation |
| CO₂ concentration monitoring inside educational buildings as a strategic tool to reduce the risk of Sars-CoV-2 airborne transmission | Observational study | 9 schools (11 classrooms): 2 pre-school, 5 primary and 4 first-level secondary classrooms in Apulia Region (South of Italy | Pilot study: CO2 surveillance activity for 3 weeks | Only natural ventilation was not effective to guarantee adequate air exchanges🡪further strategies based on heating, ventilation, air conditioning systems, air cleaner  Indoor CO_2_ monitoring is a practical tool for COVID-19 transmission risk.  All classrooms showed inadequate ventilation conditions (>700 ppm) | Natural ventilation |
| Indoor Environmental Quality and Effectiveness of Portable Air Cleaners in Reducing Levels of Airborne Particles during Schools' Reopening in the COVID-19 Pandemic | Observational study | 7 schools: 1 pre-primary and 6 secondary school (19 different classroom) in Castilla la Mancha region (Central–Southern Spain) | 3 sampling campaigns: temperature, relative humidity, CO2, particulate matter | Rooms with open windows and 1 unit of PACs had insufficient levels of safe particle concentration.  CO2 concentrations  were significantly associated with the number of children in the classroom.  Classrooms only with air purifier and natural ventilation with low levels of CO2 and adjusting the opening of windows: particle concentration levels were lower than in the case of closed or partially closed windows | Natural ventilation and PACs (portable air cleaners) |
| Effect of Infectious Disease Risk Management on Indoor Environmental Quality in Lecture Rooms: Current Performance and Future Considerations | Observational study | 10 university lecture rooms | Indoor environmental quality evaluation: CO2, occupancy, ventilation | No statistical differences between data from natural and mechanical ventilation.  Mechanical ventilation and hybrid ventilation are more likely to suppress viruses than natural ventilation.  There is a need to retrofit lecture rooms with more advanced natural or mechanical ventilation | Natural and mechanical ventilation |
| Ventilation during COVID-19 in a school for students with intellectual and developmental disabilities (IDD) | Observational study | Mary Cariola Center school for students with intellectual and developmental disabilities.  (100 classrooms) in New York (USA) | CO2 measures, ACH calculation, collection of COVID-19 cases over 20 months | Rooms with HVAC systems with MERV-13 filters had a lower incidence of COVID-19 positive PCR tests, compared to MERV-11.  No statistically significant correlation was found between ACH and COVID-19 cases | Air cleaners and filters (MERV11, MERV13) |
| COVID-19 MITIGATION IN A K-12 SCHOOL SETTING: A CASE STUDY OF AVENUES: THE WORLD SCHOOL IN NEW YORK CITY | Observational study | Private school in New York (USA) | IAQ measurements and collection of COVID-19 cases | Reduction of prevalence of COVID-19 through different mitigation measures | Air cleaners and filters |
| Associations between illness-related absences and ventilation and indoor PM_2.5_ in elementary schools of the Midwestern United States | Observational study | 31 schools (144 classrooms) in Midwestern United States (Nebraska and Iowa), USA | Ventilation rates, indoor PM2.5 concentration, CO2 concentrations, student attendance records | Greater ventilation significantly reduced illness-related absences.  Higher concentrations of indoor PM_2.5_ were associated with increased illness-related absences.  Mechanically ventilated classrooms had relatively lower indoor PM_2.5._  Every 1 L/s per person increase in ventilation rate 🡪 5.59 decrease in absent days per year (0.15% increase in the annual daily attendance rate). Every additional 1 μg/m^3^ of indoor PM_2.5_ 🡪 7.37 increase in days with absences per year (0.19% decrease in the annual daily attendance rate) | Mechanical ventilation |
| Ventilation and thermal conditions in secondary schools in the Netherlands: Effects of COVID-19 pandemic control and prevention measures | Observational study | 31 classrooms in 11 Dutch secondary schools | Survey to monitor indoor and outdoor CO2 concentration and air temperature, technical questionnaire and interview | With windows and doors kept open ventilation standards were not achieved.  Reducing occupancy can increase the ventilation rate per student in the classrooms | Natural and mechanical ventilation |
| The effect of post-COVID-19 ventilation measures on indoor air quality in primary schools | Observational study | 252 classrooms across 27 Dutch primary schools in Limburg province, Netherlands | Measurements of CO2 levels and fine particle concentration | Mechanically ventilated classrooms perform better with respect to CO2 and fine particle levels | Natural and mechanical ventilation |
| Evaluating the impact of air purifiers and window operation upon indoor air quality - UK nurseries during Covid-19 | Observational study | 3 nurseries in London, UK | Air quality sensor and passive sampling methods: PM2.5, volatile organic compounds, aldehydes | Mean reduction rate of PM2.5 by using air purifier: 63% with closed window and 46% with open window.  Air purifiers can be effective at reducing PM2.5 when combined with proper window openings | Natural ventilation and air purifiers |
| Long-term filter efficiency of mobile air purifiers in schools | Observational study | 2 rooms: seminar room at Goethe University  Frankfurt, and high school Kaiserin-Friedrich-Gymnasium (KFG) in Germany | Monitoring of devices and particles decay | With 1 infected person in the classroom:  the additional use of air purifiers reduced the number of inhaled particles viruses at the end of the day by a factor of 2.65, compared to without air purifiers | HEPA filters |
| Assessment of ventilation rates inside educational buildings in Southwestern Europe: Analysis of implemented strategic measures | Observational study | 14 classrooms: 8 on Azurém Campus (Guimaraes, Portugal) and 6 on the Fuentenueva Campus (Granada, Spain) | In situ measurements, ventilation rate using the CO_2_ decay method, probability of infection risk using the Wells-Riley equation | Ventilation rates: between 2.9 and 20.1 air change per hour (ACH) for natural cross ventilation, 2.0 to 5.1 ACH for single-sided ventilation and 1.8 to 3.5 for mechanically ventilated classrooms.  The probability of risk was less than 1% in almost all classrooms.  Natural ventilation (door and windows opening) can provide sufficient air renewal maintaining a low risk of infection | Natural and mechanical ventilation |
| Assessment of indoor air quality and risk of COVID-19 infection in Spanish secondary school and university classrooms | Observational study | Secondary school and University in Toledo, Spain | Outdoor and indoor CO_2_ levels, relative transmission risk of COVID-19 using the web app *COVID Risk^airborne^* | Mechanical ventilation: better ventilation conditions and better at maintaining an acceptable risk of transmission, especially when the use of masks was considered | Natural and mechanical ventilation |

**Table 3: OBSERVATIONAL STUDY AND MATHEMATICAL MODELING STUDY**

| **Title** | **Study type** | **Setting** | **Methods** | **Main results** | **Ventilation type** |
| --- | --- | --- | --- | --- | --- |
| Seasonal variation in airborne infection risk in schools due to changes in ventilation inferred from monitored carbon dioxide | Observational study and mathematical modeling study | 11 schools (8 primary, 3 secondary schools): 45 classrooms in the UK | CO2 measurements and risk modeling based on Wells-Riley model for 5 days in January and July 2018 | The data highlights  January being twice as risky as July for COVID-19 transmission. Classrooms with more active-vocal activities:  the airborne risk of transmission increases  by a factor of nearly 5 | Hybrid ventilation (natural + mechanical) |
| High energy efficiency ventilation to limit COVID-19 contagion in school environments | Observational study and mathematical modeling study | 2 high schools (4 classrooms) in Treviso and Cremona (Italy) | Indoor-outdoor CO2 measurements and actual changes per hour ACH | With natural ventilation, frequent and short periods of ventilation reduce CO2 concentration, but the recommended concentration is not guaranteed all the time. Dangerous in the presence of an asymptomatic person.  Infection risk >1%.  Mechanical ventilation reduced risk from 23% (8 L s^−1^ person^−1^) to 7.2% (32 L s^−1^ person^−1^) with additional filtration (efficiency 95%): 0.38%.  Reduction  of indoor viral concentration and consequently of the infection risk by a strong mechanical ventilation. High ventilation rates permit limited facemask use to have R0 <1 | Natural ventilation (different ventilation scenarios) and mechanical ventilation (different ACH) |
| Estimating aerosol transmission risk of SARS-CoV-2 in New York City public schools during reopening | Observational study and mathematical modeling study | 19 elementary schools (101 classrooms: 86 during heating season, 69 during cooling season, 54 in both) | IAQ survey (CO2, temperature, relative humidity) and Wells-Riley equation | COVID-19 transmission risk was lower in schools with mechanical ventilation. Mask wearing was associated with lower risk of transmission | Natural and mechanical ventilation (only 37% of schools) |
| Natural ventilation strategy and related issues to prevent coronavirus disease 2019 (COVID-19) airborne transmission in a school building | Observational study and mathematical modeling study | 1 elementary school (3 classrooms) in South Korea | Measurement of CO_2_ decay by cross vs single-sided ventilation with 0%, 15%, 30% and 100% window opening ratio (when unoccupied classroom) + Wells-Riley model | Natural ventilation not enough: the infection probability can be reduced by about 4 times when a mask was worn under the same ventilation rate conditions  + better cross ventilation compared to single sided ventilation.  Without ventilation: infection risk >1% even with mask and exposure time of 0.5h.  Infection risk <1% with cross ventilation without mask with 30% window opening and 1 h exposure. Infection risk of <1% with single sided ventilation only with masks and exposure time of max. 1 h | Natural ventilation (cross-sided and single ventilation) |
| Mitigating aerosol infection risk in school buildings: the role of natural ventilation, volume, occupancy and CO₂ monitoring | Observational study and mathematical modeling study | 1 high school (1 classroom) in Northern Italy | CO_2_ measurements with real occupants + comprehensive risk mitigation strategy | Only natural ventilation during breaks is not sufficient. It is necessary to add other measures: microphone (20% risk reduction (without masks) and almost 40% with surgical masks and increasing the classroom size (risk reduced by half) | Natural ventilation (during breaks) and mechanical ventilation + model with-without masks and microphone for the teacher |
| Study on Droplet Dispersion Influenced by Ventilation and Source Configuration in Classroom Settings Using Low-cost Sensor Network | Observational study and mathematical modeling study | University classroom: 2 types: air handling unit (AHU) and fan coil unit (FCU), University of Minnesota, USA | Monitoring of airborne particle concentration, simulation | The respiratory droplet source should be in the location such that the particle dispersion opposes the ventilation flow.  The AHU and FCU should be both turned on during class hours | Mechanical ventilation |
| Mask Use and Ventilation Improvements to Reduce COVID-19 Incidence in Elementary Schools- Georgia | Survey in schools and mathematical modeling study | 69 elementary and K-5 schools in Georgia, USA | Survey in schools on prevention strategies: increased natural ventilation, filtration, masks, physical distancing, desks barriers, cohort size | Improving ventilation strategies resulted in 35% lower incidence of COVID-19.  48% reduction with increased natural ventilation and air filtration/purification and 37% reduction with masks (students and staff) | Increased natural ventilation, air filtration/purification, mask use |
| Analysis of IAQ in classrooms during COVID-19 pandemic and the effect of window ventilation and air cleaners depending on season | Observational study and mathematical modeling study | 2 classroom level 5-12 (students aged from 11-12 to 18) in a city in Germany | CO2 measurements, reduction strategies, 90 minutes simulated lesson with virus particles (decay of particle number concentration) without students | The combination of air cleaners and window ventilation reduces particle number concentration.  Air cleaner can minimize the particle number concentration regardless of season and ventilation behavior: reduction of inhaled particles by >50% during 90 min lesson.  The same result is achieved with exclusive windows ventilation only if multiple windows are open simultaneously.  Measures beyond natural ventilation are necessary | Natural ventilation with/without air cleaners |
| Air quality monitoring device to mitigate the spread of COVID-19 in educational buildings | Observational study and mathematical modeling study | 3 University classrooms at the Antonio Narino University in Colombia, Bogota | Measurement of CO2, PM2.5 and estimation of infection risk for COVID-19 | Indoor CO2 concentrations and the risk of COVID-19 infection are influenced by the activity type and the number of windows open. Talking and moving activities are the ones which cause higher indoor CO2 and transmission risk.  Exams, lectures, and teamwork have a low COVID-19 transmission risk | Natural ventilation |
| COVID-19 Infection Risk Assessment in a Kindergarten Utilizing Continuous Air Quality Monitoring Data | Observational study and mathematical modeling study | Kindergarden in Taipei, Taiwan | CO2 monitoring on site + scenario analysis for improvement measures (Wells-Riley equation) | 78% to 92% of the school hours had indoor CO_2_ level higher than the standard norms.  Infection risk in the classes was about 10%.  With one infected individual, the infection could be transmitted to 2.13–4.23 susceptible persons during the peak hours (highest CO_2_ concentration).  To lower the transmission risk: reduce the number of occupants, the duration of their stay, and indoor CO₂ concentration (improve ventilation systems) |  |
| Air change rates and infection risk in school environments: Monitoring naturally ventilated classrooms in a northern Italian | Observational study and mathematical modeling study | 3 schools: 2 primary schools (A and B) and a secondary school (C) in Milan, Italy | CO2 measurements, indoor-outdoor air temperature, windows opening configuration, air change rates calculation (Wells-Riley model) | The bigger the window opening ratio, the more the air change rates increase, the lower the probability of infection.  European recommendation for air renewal: achieved by a window opening area of at least 1.5 m.  The scenario with an infected teacher has higher risk of infection compared to the scenario with an infected student | Natural ventilation |
| Assessment of natural ventilation strategy to decrease the risk of COVID 19 infection at a rural elementary school | Observational study and mathematical modeling study | 9 classrooms of a public elementary school (Los Zumacales) in a rural area of Castilla y Leon (North-Western Spain) | Measurement of CO2, temperature, relative humidity, level of thermal comfort, decay CO2 concentration method, REHVA methodology (infection risks) | Only one classroom showed a value lower than 3, indicating poor ventilation.  The probability of infection in all the cases studied was less than 14%.  Necessary to open windows/doors at the end of each lecture | Natural Ventilation |
| Airborne infection risk in classrooms based on environment and occupant behavior measurement under COVID-19 epidemic | Observational study and mathematical modeling study | 3 typical classrooms of a primary school in  Tianjin, China | On site measurements: CO2 measurements, occupant area per person and activity level (survey) and Wells-Riley model to predict infection risk | The infection risk transmission in the three classrooms was influenced by the concentration of people indoors, by the occupational area | Air cleaners and air purifiers |
| The relationship between ventilation rates in schools and the indoor airborne transmission potential of COVID-19 | Observational study and mathematical modeling study | 10 naturally ventilated classrooms Irish post primary schools, Dublin | CO2 sensors, calculation of ventilation rates, calculation of rate of transmission of COVID-19 (use of Wells-Riley model) | With 5 ACH: infection risk of less than 1%.  Most of all calculated rates: 2 ACH or lower.  Reduction of infection transmission risk using a mask: 7–12% to 1–5%. Over an 8-hour school day, probability of infection can be reduced from 75–100% to 18–43%, with a mask | Natural ventilation |
| Air Cleaners and Respiratory Infections in Schools: A Modeling Study Based on Epidemiologic, Environmental, and Molecular Data | Observational study and mathematical modeling study | 2 Swiss secondary school classes in Solothurn, Switzerland | Collection of environmental (CO_2_, particle concentrations), epidemiological (absences related to respiratory infections), audio (coughing), and molecular data (bioaerosol and saliva samples) | With air cleaners, mean particle concentration decreased by 77%. There were no differences in CO2 levels. Absences related to respiratory infections were 22 without air cleaners vs 13 with them.  The estimated number of respiratory infections in school would have been 19 if air cleaners had been installed throughout the study period, as compared with 36 if air cleaners had not been installed | Air cleaners and HEPA filters |
| Quantitative Assessment of Natural Ventilation in an Elementary School Classroom in the Context of COVID-19 and Its Impact in Airborne Transmission | Observational study and mathematical modeling study | 2 elementary schools in Uruguay | CO2 measurements 5 months), ventilation rates, infection risk calculations (Wells-Riley), analytical simulations for different  scenarios | Periodic ventilation is an extremely efficient strategy  for reducing transmission risk.  Ventilation rates improved from winter to spring, (decline in estimated infection risk) | Natural ventilation |
| Monitoring CO₂ concentration to control the infection probability due to airborne transmission in naturally ventilated university classrooms | Observational study and mathematical modeling study | 20 university classrooms in Pisa, Italy | CO2 measurements, Wells-Riley equation for infection risk | High CO₂ levels correlate with high risk of airborne transmission of COVID-19.  Higher class occupancy and increased activity levels lead to elevated CO₂ concentrations.  Effective FFP2 mask use is the only measure that allows for keeping  the infection probability < 1%.  Cloth or surgical masks are not sufficient to keep the infection probability below 1% for one hour of exposure, while highly effective masks are efficient in all the configurations | Natural ventilation |

**Table 4: MATHEMATICAL MODEL**

| **Title** | **Study type** | **Setting** | **Methods** | **Main results** | **Ventilation type** |
| --- | --- | --- | --- | --- | --- |
| Airborne infection risks of SARS-CoV-2 in US schools and impacts of different intervention strategies | Mathematical modeling study | 111 485 public and private schools in the USA | Scenario-based analysis and epidemiological scenario, infection risk modeling | Infection risk in 90% of the schools: >1%. Lower in July (3.85%) compared to December (6.83%).  Infection risk decreases by 16.5% by increasing ventilation rate from 2/h to 2.5/h and by 8% from 5.5/h to 6/h.  Air filtration is found to be most effective: the school average infection risk when applying MERV 13 is over 30% less than the risk levels correlating with the use of increased ventilation and hybrid learning.  It is necessary to combine intervention strategies for infection risk< 1% | Air filters: MERV 13 |
| A systematic approach to estimating the effectiveness of multi-scale IAQ strategies for reducing the risk of airborne infection of SARS-CoV-2 | Mathematical modeling study | Different indoor spaces: K-12 schools | Infection risk estimation model: Wells-Riley | Higher probability of COVID-19 transmission from teacher to students (13.2%) than student to student (3.8%).  Reduction of infection risk: 37% reduction when doubling total supply airflow rate.  100% reduction with outdoor air.  27% reduction with HEPA filter.  26% reduction when displacement ventilation.  46% reduction when partitions.  46% reduction with personal ventilation | Natural ventilation, HEPA filters, displacement ventilation, partitions, personal ventilation |
| Ventilation procedures to minimize the airborne transmission of viruses in classrooms | Mathematical modeling study | 1 high school (1 classroom) | Simulation of 5h school day | Air exchange rate <0.1/h for preventing seasonal influenza.  9.5/h and 0.8/h if the teacher is infector to prevent COVID-19 infection (60 min speaking versus through microphone). Required AER if the student is infector depends on speaking time and attendance: 0.8–3.5/h.  Long ventilation periods or high air exchange rates not always possible with natural ventilation  Mitigation solutions (voice modulation) or immunization are needed | Natural and mechanical ventilation: simulation of different scenarios (different infectors, intensity of speaking) |
| Assessing school ventilation strategies from the perspective of health, environment, and energy | Mathematical modeling study | University seminar room in Graz University of Technology in Germany | Experimental setup with empirical measurements with building performance simulation and analytic risk analysis  Infection risk calculated for n=20 | None of the ventilation strategies eliminated the transmission risk, but the hybrid and mechanical ventilation scenario provided the best compromise. However, the use of masks reduces the risk of infection transmission for all the ventilation strategies | Natural ventilation, mechanical ventilation, hybrid ventilation : 5 different scenarios |
| Estimating the nationwide transmission risk of measles in US schools and impacts of vaccination and supplemental infection control strategies | Mathematical modeling study | Primary and secondary schools in the USA | Wells-Riley approach, nationwide representative School Building Archetype (SBA) model, and Monte-Carlo simulation.  Estimates were validated with real outbreak data | Transmission risk 74 times higher for unvaccinated students, higher in high schools (median 5.8%) than in elementary schools (median 3.8%).  Highest transmission risk (median 6%) in schools with ductless systems without air filters.  Lowest transmission risk (3.7% median) in schools with ductless systems with air filters.  For unvaccinated students: using a better filter reduced transmission risk (median 45% for MERV8, 32% for MERV13, and 29% for HEPA filter), and also increasing ventilation rates (46% basic control, 38% regular, 33% advanced infection scenario) | Ventilation/purification/filtration (MERV, HEPA) |
| ENERGY PERFORMANCE AND INFECTION RISK EVALUATION OF RETROFITTED VENTILATION SYSTEMS IN TIMES OF COVID | Mathematical modeling study | Lecture room in Ireland | Simulation model | The retrofitted mechanical ventilation system significantly enhanced indoor air quality compared to natural ventilation strategies and decreased risk transmission of COVID-19 | Natural and mechanical ventilation |
| A New Tailored Approach to Calculate the Optimal Number of Outdoor Air Changes in School Building HVAC Systems in the Post-COVID-19 Era | Mathematical modeling study | Kindergarden school building (7 classrooms) in Fondi, Italy | Simulation model  (Wells-Riley equation) with 5 ventilation strategies | Increasing ventilation rates in a room is not an optimal ventilation strategy to control the infection risk. The study proposes setting a consistent low contagion risk level, rather than applying a uniform high ventilation rate | HVAC system |
| CO₂ concentration as an indicator of indoor ventilation performance to control airborne transmission of SARS-CoV-2 | Mathematical modeling study | School classroom | Mathematical modeling study applied to real-world scenarios (school classroom).  Calculations for the mean indoor CO2 concentration and the air exchange rate to control COVID-19 airborne transmission | Classroom with 20–25 students and an exposure time of 6–8h: average indoor CO2 concentration should be kept below 700 ppm. Appropriate ventilation rate is sufficient when wearing a mask in classrooms | Air cleaners and filters |
| Window Opening or Mechanical Ventilation Systems for Italian Schools? Energetic Aspects, CO₂ Concentration and Infection Risk Assessment Based on the SARS-CoV-2 Data | Mathematical modeling study | School classrooms in Padova, Torino, Roma, Napoli, Palermo, Italy | IAQ, Wells-Riley model | Mechanical ventilation reduced 3 times the risk of infection, with and without a mask, and it could outperform the effect of masks | Natural and mechanical ventilation, with or without mask |
| SARS-CoV-2 aerosol transmission in schools: the effectiveness of different interventions | Mathematical modeling study |  | Aerosol transmission model | Combined interventions  (natural ventilation, masks, and HEPA filtration) were  the most effective.  HEPA filtration combined with face masks can reduce the mean aerosol concentration  by 90%.  The universal use of surgical masks was one of the most effective interventions | Natural, mechanical ventilation, HEPA filtration, masks |
| The impact of retrofitted ventilation approaches on long-range airborne infection risk for lecture room environments: design stage methodology and application | Mathematical modeling study | Study lecture room (university classroom) in Dublin, Stockholm, Rome and Budapest | 3 stage analytical simulation methodology: natural ventilation airflow rate model, airborne infectious risk model, seasonality check | Mechanical ventilation system is very effective at suppressing the virus infection risk.  Mask can suppress this risk substantially under the same external condition.  Natural ventilation is seasonally vulnerable | Natural and mechanical ventilation, masks |
| A Monte Carlo Assessment of the Effect of Different Ventilation Strategies to Mitigate the COVID-19 Contagion Risk in Educational Buildings | Mathematical modeling study | Study classroom at the Free University of  Bozen-Bolzano, Italy | Monte Carlo method and building performance simulation | Importance of  combining different solutions (mixed-mode ventilation and masks) to limit the risk for both students and lecturers | Natural and mechanical ventilation |
| Assessment of respiratory disease infection risk and natural ventilation intervention countermeasures in teaching spaces: A campus case study | Mathematical modeling study | Shenyang Jianzhu University teaching space, China | Wells-Riley model and basic reproduction number R0 calculations | Natural ventilation is not efficient to reduce the risk of infection.  With optimization:  indoor virus infection probability can be reduced by 22.88% | Natural and mechanical ventilation |
| Ventilation strategy for simultaneous management of indoor particulate matter and airborne transmission risks-A case study for urban schools in South Korea | Mathematical modeling study | Classrooms of 13 schools in urban areas in South Korea | CO2, PM, open/closed window and doors, Wells-Riley equation | Quanta could be removed quickly with cross-ventilation with 100% of windows open.  It was not possible to meet the safe condition without wearing a mask, and all 24 susceptible students were infected | Natural ventilation |
| The effect of natural ventilation on airborne transmission of the COVID-19 virus spread by sneezing in the classroom | Mathematical modeling study | Secondary school classroom in Sanliurfa, Turkey | Computational fluid dynamics (CFD) simulation results and Eulerian-Lagrange method, a discrete phase model, and a temporary three-dimensional CFD model, 5 scenarios | Natural ventilation in the classroom on the transmission of the virus travel was negligible | Natural ventilation |
| Modeling impacts of ventilation and filtration methods on energy use and airborne disease transmission in classrooms | Mathematical modeling study | Classroom in 13 large cities: Miami, Houston, Phoenix, Atlanta, Los Angeles, San Francisco, Las Vegas, Baltimore, Albuquerque, Seattle, Chicago. Denver, Minneapolis, USA | Simulation of outdoor air ventilation rates and filtration methods, modified Wells-Riley equation and EnergyPlus building simulation | Infection probability was 29% lower with improved filtration.  (MERV 13 filters, IRF, or both) in comparison to increasing ventilation rates above 7.0 l/(person) | HVAC filtration system model |

**Table 5: REVIEWS**

| **Title** | **Study type** | **Setting** | **Methods** | **Main results** | **Ventilation type** |
| --- | --- | --- | --- | --- | --- |
| A multi-layered strategy for COVID-19 infection prophylaxis in schools: A review of the evidence for masks, distancing, and ventilation | Sytematic review | Schools |  | High-quality  masks and regular  testing are the most effective measures,  natural and mechanical ventilation reduces infection risks in classrooms by over 80%. Ventilation systems and air purifiers cannot obviate the need  for masking and physical distancing | Natural and mechanical ventilation |
| A Systematic Review on Indoor Environmental Quality in Naturally Ventilated School Classrooms: A Way Forward | Systematic review |  |  | No proper evidence to  prevent the transmission of COVID-19 inside  naturally ventilated school. Best way: fresh air after proper  filtration and mechanical ventilation | Natural ventilation |
| Ventilation in school and students' health after outbreak of COVID-19: A systematic literature review | Literature review | Primary, middle and secondary school |  | As ventilation reduces indoor air pollutants, it should be prioritized for student’s health |  |
| Indoor Air Quality and COVID-19: A Scoping Review | Scoping review | Workplaces, schools, restaurants, and public transport |  | Ventilation could be a possible effective mitigating method for COVID-19 transmission reduction |  |
| Assessing Indoor Air Quality and Ventilation to Limit Aerosol Dispersion-Literature Review | Literature review | Schools or offices |  | To limit the aerosol dispersion in indoor spaces, different mitigation measures |  |
| The Influence of Ventilation Measures on the Airborne Risk of Infection in Schools: A Scoping Review | Scoping Review | Schools |  | Ventilation is an important measure to reduce the risk of airborne infections in schools. The most important effect is to reduce the time of pathogens in the classrooms |  |
| Airborne transmission of SARS-CoV-2 in indoor environments: A comprehensive review | Comprehensive review | Schools |  | Mobile air purifiers reduce the transmission risk of COVID-9 |  |
