# Supplementary material for: Ventilation and filtration strategies to reduce respiratory infections in schools: a scoping review
Source: Public Health Rev. 2026 Jul 16;47:1608923. doi: 10.3389/phrs.2026.1608923 (PMC13454833; doi:10.3389/phrs.2026.1608923)
Supplement: Supplementary file 2 [file Supplementaryfile2.docx]

**Supplementary Material : Appendix 2 : Research Query**

- Research query used on Pubmed on 19.03.2025

("SARS-CoV-2"[Mesh] OR "COVID-19"[Mesh] OR "Respiratory Syncytial Virus Infections"[Mesh] OR "Influenza, Human"[Mesh] OR "2019 ncov"[Title/Abstract] OR "2019 Novel Coronavirus Disease*"[Title/Abstract] OR "2019 Novel Coronavirus Infection*"[Title/Abstract] OR "2019 ncov"[Title/Abstract] OR "Coronavirus Disease 2019"[Title/Abstract] OR "Coronavirus Disease 2019"[Title/Abstract] OR "COVID"[Title/Abstract] OR "COVID-19"[Title/Abstract] OR "Grippe"[Title/Abstract] OR "Human Flu"[Title/Abstract] OR "influenza*"[Title/Abstract] OR "Respiratory Syncytial Virus Infection*"[Title/Abstract] OR "rsv infection*"[Title/Abstract] OR "SARS Coronavirus 2 Infection"[Title/Abstract] OR "sars cov 2 infection*"[Title/Abstract] OR "sars cov 2 infection*"[Title/Abstract] OR "Severe Acute Respiratory Syndrome Coronavirus 2 Infection*"[Title/Abstract] OR "viral respiratory tract infection*"[Title/Abstract] OR "VRS"[Title/Abstract] OR "winter respiratory infection*"[Title/Abstract] OR "coronavirus disease 19"[Title/Abstract] OR "coronavirus disease 19"[Title/Abstract]) AND ("Air Pollution, Indoor"[Mesh] OR "Ventilation"[Mesh] OR "Environment, Controlled"[Mesh:NoExp] OR "Air Conditioning"[Mesh] OR "indoor climate"[Title/Abstract] OR "Indoor Air"[Title/Abstract] OR "indoor environment*"[Title/Abstract] OR "Room air"[Title/Abstract] OR "natural ventilation"[Title/Abstract] OR "air circulation"[Title/Abstract] OR "air recirculation"[Title/Abstract] OR "adequate ventilation"[Title/Abstract] OR "air filtration"[Title/Abstract] OR "room ventilation"[Title/Abstract] OR "indoor ventilation"[Title/Abstract]) AND ("Schools"[MeSH Terms] OR "Universities"[Mesh] OR "school*"[Title/Abstract] OR "University"[Title/Abstract] OR "Universities"[Title/Abstract]) Filters: in the last 5 years, Classical Article, Clinical Study, Clinical Trial, Observational Study, Randomized Controlled Trial, Review, Systematic Review, English, French, Italian

- Research query used on Embase on 19.03.2025

('viral respiratory tract infection'/exp OR 'severe acute respiratory syndrome coronavirus 2'/exp OR 'coronavirus disease 2019'/exp OR 'respiratory syncytial virus infection'/exp OR 'influenza'/exp OR '2019 novel coronavirus disease':ti,ab,kw OR '2019 novel coronavirus infection':ti,ab,kw OR '2019 ncov':ti,ab,kw OR 'coronavirus disease 2019':ti,ab,kw OR 'covid':ti,ab,kw OR 'covid-19':ti,ab,kw OR 'grippe':ti,ab,kw OR 'human flu':ti,ab,kw OR 'influenza*':ti,ab,kw OR 'respiratory syncytial virus infections':ti,ab,kw OR 'rsv infection*':ti,ab,kw OR 'sars coronavirus 2 infection':ti,ab,kw OR 'sars cov 2 infection*':ti,ab,kw OR 'severe acute respiratory syndrome coronavirus 2 infection':ti,ab,kw OR 'viral respiratory tract infection':ti,ab,kw OR 'vrs':ti,ab,kw OR 'winter respiratory infections':ti,ab,kw OR 'coronavirus disease 19':ti,ab,kw) AND ('indoor air pollution'/exp OR 'air conditioning'/exp OR 'air filtration'/exp OR 'natural ventilation':ti,ab,kw OR ((('indoor' OR 'room') NEAR/2 ('climate' OR 'air' OR 'environment*' OR 'ventilation')):ti,ab,kw) OR (('air' NEAR/2 ('circulation' OR 'recirculation' OR 'filtration')):ti,ab,kw) OR 'adequate ventilation':ti,ab,kw) AND ('school'/exp OR 'university'/exp OR 'school*':ti,ab,kw OR 'university':ti,ab,kw OR 'universities':ti,ab,kw) AND Présentation PowerPoint (2020:py OR 2021:py OR 2022:py OR 2023:py OR 2024:py OR 2025:py) AND ('article'/it OR 'review'/it) Présentation PowerPoint AND ('case study'/de OR 'clinical article'/de OR 'intervention study'/de OR 'major clinical study'/de OR 'observational study'/de OR 'randomized controlled trial'/de OR 'systematic review'/de) AND ('article'/it OR 'review'/it)

- Research query used on Web of Science on 19.03.2025

(TS =("2019 ncov" OR "2019 Novel Coronavirus Disease*" OR "2019 Novel Coronavirus Infection*" OR "2019 ncov" OR "Coronavirus Disease 2019" OR "Coronavirus Disease 2019" OR "COVID" OR "COVID-19" OR "Grippe" OR "Human Flu" OR "influenza*" OR "Respiratory Syncytial Virus Infection*" OR "rsv infection*" OR "SARS Coronavirus 2 Infection" OR "sars cov 2 infection*" OR "sars cov 2 infection*" OR "Severe Acute Respiratory Syndrome Coronavirus 2 Infection*" OR "viral respiratory tract infection*" OR "VRS" OR "winter respiratory infection*" OR "coronavirus disease 19" OR "coronavirus disease 19")) AND (TS =("indoor climate" OR "Indoor Air" OR "indoor environment*" OR "Room air" OR "natural ventilation" OR "air circulation" OR "air recirculation" OR "adequate ventilation" OR "air filtration" OR "room ventilation" OR "indoor ventilation")) AND (TS =("school*" OR "University" OR "Universities")) AND (TS =("indoor climate" OR "Indoor Air" OR "indoor environment*" OR "Room air" OR "natural ventilation" OR "air circulation" OR "air recirculation" OR "adequate ventilation" OR "air filtration" OR "room ventilation" OR "indoor ventilation")) AND (TS =("school*" OR "University" OR "Universities"))
