# Supplementary material for: Ventilation and filtration strategies to reduce respiratory infections in schools: a scoping review
Source: Public Health Rev. 2026 Jul 16;47:1608923. doi: 10.3389/phrs.2026.1608923 (PMC13454833; doi:10.3389/phrs.2026.1608923)
Supplement: Supplementary file 3 [file Supplementaryfile3.docx]

**Supplementary Material : Appendix 3**

|  | **Title** | **Author(s) names** | **Date of publication** | **Methods** | **Aim** | **Study period of investigation** | **Type of indoor setting** | **City** | **Main results** | **Natural VS mechanical ventilation** | **Limitations** |
| --- | --- | --- | --- | --- | --- | --- | --- | --- | --- | --- | --- |
|  | Analysis of IAQ in classrooms during COVID-19 pandemic and the effect of window ventilation and air cleaners depending on season | Finn Felix Duill, Florian Schulz, Aman Jain, Nils Paucke, Berend van Wachem,  Frank Beyrau | 2025 | CO2 measurements + reduction strategies + 90 min simulated lesson with virus particles (decay rates of PNC (particle number concentration) for different scenarios) (no students present)  Observational study and mathemtical modeling study | To measure the CO2 level and the effect of window ventilation and air cleaners | June-July 2022- March 2023 | 2 classrooms level 5-12 (students aged from 11-12 to 18) | City in central Germany | The average CO2 concentration is double in winter compared to summer (initial concentration is higher, insufficient intensity and long duration between ventilation phases).  The combination of air cleaners and window ventilation reduces PNC, and their interaction is influenced by external conditions and ventilation type.  Air cleaners can minimize PNC regardless of season and ventilation behavior: reduction of inhaled particles by >50% during 90 min lesson.  The same result is achieved with exclusive windows ventilation only if multiple windows are open simultaneously.  Measures beyond natural ventilation are necessary | NATURAL VENTILATION WITH AND WITHOUT AIR CLEANERS  No HVAC  1 air cleaner and 5 windows.  No individual in the room during the measurements for PNC (90 min lesson).  Aerosol generator for 15 minutes (homogeneously).  Ventilation: with/without windows, with/without door open, tilt ventilation, shock ventilation | -PNC values are not universally applicable due to: ventilation area, wind speed and direction, temperature difference indoor-outdoor.  -Use of large air cleaners, which is different compared to small ones.  -Viral particles have been homogenized in the room and measurements are made without children present. Even without ventilation strategy, there is a continuous reduction in the number of viral particles |
|  | Assessing school ventilation strategies from the perspective of health, environment, and energy | Fatos Pollozhani, Robert S. McLeod, Christian Schwarzbauer, Christina J. Hopfe | 2024 | Experimental setup with empirical measurements with building performance simulation and analytic risk analysis.  Infection risk calculated for n=20  Mathematical modeling study | To evaluate the functionality of different natural and mechanical ventilation strategies through simulations of 5 ventilations scenarios | October-April  May-June | University seminar room (fairly 30 participants) | Graz University of Technology in Germany | None of the ventilation strategies eliminated the transmission risk, but the hybrid and mechanical ventilation scenario provided the best compromise. However, the use of masks reduces the risk of infection transmission for all the ventilation strategies.  Mechanical ventilation has the second lowest risk, with or without mask + it is not dependent on seasons.  Then, natural ventilation (tilt or purge) (dependent on seasons). | MECHANICAL AND HYBRID VENTILATION VS NATURAL VENTILATION  Mechanical extract ventilation (MEV) and 5 tilt and turn windows.  Risk of infection analyzed for n=20.  -Base case:  All windows and doors closed.  CO2 higher than other case scenarios.  Higher viral concentration for n=20.  Probability of 100% that at least 1 person would become infected during 8h exposure.  With FFP2 (all): 27% risk.  For Jan and June.  -Mechanical ventilation MPIC-MEV:  Risk of 47% without masking, 4% with masking. For Jan and June.  -Air handling unit-heat recovery  57% no mask, 5% mask. For Jan and June.    -Natural ventilation (5 tilt windows)  42% no mask, 3% mask (12 Jan); 72% no mask, 7% mask (30 June).  - Natural (purge) ventilation  (fully opened windows every 20 min for 4 min during October-April, from May-June for 15 mins)  59% no mask, 5% mask (Jan 12). Same in summer due to extended summer purge duration time.  Natural ventilation very dependent on outdoor air temperature | -No hood capture effects (direct extraction of CO2) and displacement effects were taken into account.  -Assumptions: perfect installation, maintenance, performance of mechanical systems, timely opening of windows.  -Immunologically naïve population (actual infection may be lower).  -Short-range transmission is not included |
|  | Air quality monitoring device to mitigate the spread of COVID-19 in educational buildings | Diego Quiroga, Sergio Diaz and Homero F. Pastrana | 2024 | Measurement of CO2, PM2.5 and estimation of infection risk for COVID-19.  Observational study and mathematical modeling study | To build an Air Quality Monitoring Device (AQMD) that measures and analyzes the levels of CO2 and particulate matter to mitigate the transmission risk of COVID-19 |  | 3 University classrooms (Antonio Narino University) in Colombia Bogota.  18-31 students per class | Bogota | Indoor CO2 concentrations and the risk of COVID-19 infection are influenced by the activity type and the number of windows open. The number of students is not significant for the indoor CO2 concentration levels because the number of students in the scenario (18 to 31) is relatively small.  Talking and moving activities are the ones which cause higher indoor CO2 and transmission risk.  Exams, lectures, and teamwork have a low COVID-19 transmission risk  Opening windows is an effective measure to reduce the risk of infection transmission (here for COVID-19).  Lectures with 22-23 students and four open windows had a lower CO2 concentration and a lower transmission risk compared to lectures with 21 students and windows closed | NATURAL VENTILATION | Threshold calculation for CO2:  -uncertainties (virus exhalation rates);  -assumption about  social distance (eliminate close proximity aerosol  and droplets);  -it does not consider fomite transmission |
|  | Room HVAC Influences on the Removal of Airborne Particulate Matter: Implications for School Reopening during the COVID-19 Pandemic | Ali Mohammadi Nafchi, Vincent Blouin, Nigel Kaye, Andrew Metcalf, Katie Van Valkinburgh  and Ehsan Mousavi | 2024 | Measurements and analytical calculations.  Intervention study | To examine the effect of ventilation type, airflow rates, and filtration on IAQ in 5 different classrooms |  | 5 classrooms of a university campus (Clemson University) | USA | Classrooms with central HVAC system responded better and faster for indoor air contamination compared to the ones with only fan coil units. Increasing the ventilation rate without improving filtration is inefficient.  Air filters are needed.  3 mechanisms: ACH, filtration, surface deposition.  For classrooms with limited resources, mitigation measures are needed | HVAC system:  Room A: 2 fan coil units + MERV-8.  Room B: mechanical ventilation.  Room C: central ventilation system.  Room D + E: HVAC system |  |
|  | A New Tailored Approach to Calculate the Optimal Number of Outdoor Air Changes in School Building HVAC Systems in the Post-COVID-19 Era | Diana D’Agostino, Martina Di Mascolo, Federico Minelli and Francesco Minichiello | 2024 | Simulation model  (Wells-Riley equation)  Mathematical modeling study | To define the optimal ventilation strategy HVAC to reduce the infection risk |  | Kindergarden school building | Central Italy (Fondi). | Increasing ventilation rates in a room is not an optimal ventilation strategy to control the infection risk. The study proposes setting a consistent low contagion risk level in all rooms, rather than applying a uniform high ventilation rate | HVAC system:  5 ventilation strategies, 7 classrooms  1.WHO approach:  Minimum ventilation flow of 10 L/s per person. 10% risk of infection. Very similar to IMD approach.  2.ACDCP Approach:  5 outdoor ACH (constant). The risk of infection is 5%.  3.Gradual increase in ventilation approach parametric analysis:  From 1 ACH to 10 ACH.  For ventilation rates lower than 5-6 ACH, significant differences in infection risk are present from one scenario to another. Increasing the ventilation rate means lowering the infection risk only by little.  Gradually increasing ventilation rates in a room does not represent an optimal ventilation strategy to control infection risk.  4.IMD Approach:  two fixed values of outdoor ACH: 2.5 ACH and 1.5 ACH. 10% risk infection in classrooms.  5.Controlled Maximum Infection Risk in all rooms:  3 infection risk thresholds of 20%, 15% and 10%.  For 20% infection risk: resulting outdoor ACH values of 1.2 h−1.  For 15% infection risk classrooms: ventilation rate equal to 1.5 h−1.  For 10% infection risk  Classrooms need a ventilation rate equal to 2.3 ACH.  This investigation limits the risk to 10% (this is the lowest attained in classrooms  when applying the IMD and WHO approaches)🡪 optimal one | -Assumptions of the method: complete and ideal air mixing.  -It does not consider the actual configuration of spaces and air supply and extraction devices.  - Increase in the outdoor ACH (and therefore the increase in the size of the air  handling units and air distribution systems).  -Assumption of the steady-state condition |
|  | COVID-19 Infection Risk Assessment in a Kindergarten Utilizing Continuous Air Quality Monitoring Data | Chung-Yen Chen, Jia-Kun Chen, Chia-Pin Chio, Pau-Chung Chen,  Ta-Chen Su, and Chang-Chuan Chan | 2024 | CO2 monitoring on site + scenario analysis for improvement measures (Wells-Riley equation)  Observational study + Mathematical modeling study | To explore different interventions for improving ventilation  systems | 24 August-10 November 2021 | Kindergarden (kids aged 2-6)  30 participants (children and teachers).  The  students were classified by age: those aged 2–3, 3–4, 4–5,  and 5–6 years belong to Classes A, B, C, and D | Taipei, Taiwan | 78% to 92% of the school hours had indoor CO_2_ level higher than the standard norms.  Infection risk in the classes was about 10%.  With one infected individual, the infection could be transmitted to 2.13–4.23 susceptible persons during the peak hours (highest CO_2_ concentration).  To lower the transmission risk: reduce the number of occupants, the duration of their stay, and indoor CO₂ concentration (improve ventilation systems) |  | -Assumption that CO2 exhalation rates are not significantly different between children and adults.  -Wells–Riley model assumption: indoor air + aerosols are well mixed +  steady state (may not be held in larger rooms or those with irregular shapes).  -Failed to consider the difference in height  between children and adults and its influence on  the CO2 concentration data |
|  | Effect of Infectious Disease Risk Management on Indoor Environmental Quality in Lecture Rooms: Current Performance and Future Considerations | Adam C. O Donovan, Fergus Delaney, Taina Ouvrard, Paol Geoffroy and Paul D. O Sullivan | 2024 | Indoor environmental quality evaluation: CO2, occupancy, ventilation  Observational study | To present an indoor environmental quality evaluation of 10 university lecture rooms before, during and after changes in ventilation systems | Pre covid: 2013-2014.  Covid: 2021.  The post-COVID-19 data | 10 university lecture rooms | Europe | No statistical differences between data from natural and mechanical ventilation.  Mechanical ventilation and hybrid ventilation are more likely to suppress viruses than natural ventilation.  There is a need to retrofit lecture rooms with more advanced natural or mechanical ventilation | NATURAL AND MECHANICAL VENTILATION | Natural ventilation classroom limitation to ventilate adequately because of the design |
|  | Indoor Environmental Quality and Effectiveness of Portable Air Cleaners in Reducing Levels of Airborne Particles during Schools' Reopening in the COVID-19 Pandemic | Florentina Villanueva, Fátima Felgueiras, Alberto Notario, Beatriz Cabanas  and Marta Fonseca Gabriel | 2024 | 3 sampling campaigns: temperature, relative humidity, CO2, particulate matter  Observational study | To assess indoors  environmental quality and test the effectiveness of portable air cleaners (PACs) for reducing airborne  particles during the period after the lockdown | September to 22 December 2020 | 7 schools: 1 pre-primary and 6 secondary school (19 different classroom) | Castilla la Mancha region (Central–Southern Spain) | Rooms with open windows + 1 unit of PACs had insufficient levels of safe particle concentration.  CO2 concentrations  were significantly associated with the number of children in the classroom.  Classrooms only 1 air purifier + natural ventilation with low levels of CO2 and adjusting the opening of windows: particle concentration levels were lower than in the case of closed or partially closed windows | NATURAL VENTILATION +PORTABLE AIR CLEANER | Possible bias:  -Mandatory use of masks during the lesson  (not for preschool children);  -Infection control (frequent hand hygiene and cleaning of surfaces);  -Maximum physical distance (1.5 m distance at least)  -Limited number of classrooms (mainly pre-school classroom).  -3 different campaigns (September to December).  -The methodology did not include the measurement of the concentration of outdoor particulate matter.  -Only 1 PAC was used per classroom (could be insufficient to improve air quality).  -Children of different ages are exposed to significantly different environmental conditions |
|  | Ventilation during COVID-19 in a school for students with intellectual and developmental disabilities (IDD) | Martin S. Zand, Samantha Spallina, Alexis Ross, Karen Zandi,  Anne Pawlowski, Christopher L. Seplaki, Jonathan Herington, Anthony  M. Corbett, Kimberly Kaukeinen, Jeanne Holden-Wiltse, Edward G. Freedman,  Lisette Alcantara, Dongmei Li, Andrew Cameron, Nicole Beaumont, Ann Dozier,  Stephen Dewhurst,John J. Foxe | 2024 | CO2 measures, ACH calculation, collection of COVID-19 cases over 20 months  Observational study | To examine the correlation between classroom ventilation  and exposure to CO2 with the incidence of COVID-19 | August  2021-August 2022 | Mary Cariola Center school for students (3-21 yo) with IDD.  100 classrooms (3 buildings) | New York (USA) | Rooms with HVAC systems with  MERV-13 filters had a lower incidence of COVID-19 positive PCR tests, compared to MERV-11.  No statistically significant correlation was found between room ACH and COVID-19 case.  Windows remained closed in all classrooms  during the measurements | AIR CLEANERS + filters | -Air flow calculations may underestimate required air flow and recommended ACH (it does not account for re-mixing of  outside air (20%) and recirculated air (80%) used by the HVAC systems)  -Bias masking: only 30% of the students + all of the professional staff and teachers |
|  | COVID-19 MITIGATION IN A K-12 SCHOOL SETTING: A CASE STUDY OF AVENUES: THE WORLD SCHOOL IN NEW YORK CITY | Ananya Iyengar, Steve Hanon, Richard Bruns, Paula Olsiewski, and Gigi Kwik Gronvall | 2024 | IAQ measurements and collection of COVID-19 cases  Observational study | To examine indoor air quality (IAQ) measures, testing and tracing mechanisms,  masking policies, ventilation procedures, and other interventions | March 2020 to January 2023 | Private school in New York: age 2-18.  1,700 students for each of the 2019-2020  and 2020-2021 school years | New York (USA) | Reduction of prevalence of COVID-19 through different mitigation measures | AIR CLEANERS + FILTERS | Bias: vaccines, masking, social distancing |
|  | Effect of portable HEPA filters on COVID-19 period prevalence: an observational quasi-interventional study in German kindergartens | Timo Falkenberg, Felix Wasser, Nicole Zacharias, Nico Mutters,  Thomas Kistemann1 | 2023 | Quasi-interventional design: observational study follows an intervention design with the intervention group using HEPA filters and the control group not | To evaluate the effect of HEPA filters on the  COVID-19 (omicron wave) infection  prevalence in kindergartens | 2021-2023 | 32 (10 intervention, 22 control) kindergartens in Rhineland  Palatinate (Germany)  2360 children (663 intervention, 1697 control) | Rhineland  Palatinate (Germany) | Control group: 186 per 1000 children Intervention group: 372 per 1000 children  HEPA filters can significantly reduce the  viral concentration in the classroom air, but not for COVID-19 prevalence in the selected kindergartens in Germany (contagion via direct face-to-face air exchange, and the contaminated air does not pass through the filter prior to air exchange between children).  Use of HEPA filters leads  to a reduction in preventive behavior (sense of security) | AIR CLEANERS + HEPA FILTERS | Possible bias: prevention strategies, use of mask, ventilation and surface decontamination frequency and group intermixing |
|  | The relationship between ventilation rates in schools and the indoor airborne transmission potential of COVID-19 | Seamus Harrington, Mark Mulville & Spyros Stravoravdis | 2023 | CO2 sensors + calculation of ventilation rates + calculation of rate of trasmission of COVID-19 (use of Wells-Riley model  Observational study + mathematical modeling study | To measure the  performance of 10 classrooms considering (a) the  ventilation capacity; (b) the probability of airborne disease  transmission  (c) the reduction of transmission risk associated with mask wearing by  all occupants | 6-week period during the heating season | 10 naturally ventilated classrooms in Ireland (Irish post primary schools, students aged 13-18 yo) | Dublin, Ireland | The designed ventilation rate is rarely achieved.  The probability of infection rises as ACH reduces. With 5 ACH: infection risk of less than 1%.  The majority of all calculated rates were 2 ACH or lower (School A – 71%; School B – 86%) = significant infection risk.  Reduction of infection transmission risk through the use of a face mask.  During specified class durations in settings with sub-optimal ventilation rates, the probability of infection can be reduced from 7–12% to 1–5%. Over an 8-hour school day, probability of infection can be reduced from 75–100% to 18–43% | NATURAL VENTILATION: windows and doors remain open for the duration of the school day | -Limitations of the Wells-Riley model:  assumes steady-state conditions and requires measurement of outdoor air supply rates.  -Bias: students and staff wear masks all day long |
|  | CO₂ concentration as an indicator of indoor ventilation performance to control airborne transmission of SARS-CoV-2 | Sowoo Park, Doosam Song | 2023 | Scenario for the CO2 concentration-based infection rate control for COVID-19: mathematical calculations  Mathematical modeling study | To calculate the mean indoor CO2 concentration and the air exchange rate to control COVID-19 airborne transmission |  |  |  | Classroom with 20–25 students and an exposure time of 6–8h: average indoor CO2 concentration should be kept below 700 ppm. Appropriate ventilation rate is sufficient when wearing a mask in classrooms | AIR CLEANERS + FILTERS |  |
|  | Air change rates and infection risk in school environments: Monitoring naturally ventilated classrooms in a northern Italian urban context | S. Ferrari, T. Blazquez, R. Cardelli, E. De Angelis, G. Puglisi, R. Escandon, R. Suàrez | 2023 | CO2 measurements, indoor-outdoor air temperature, windows opening configuration, air change rates calculation (Wells-Riley model)  Observational study + mathematical modeling study | To calculate the estimation of real air change rates through the transient mass balance equation method, and the individual infection risk via the Wells-Riley equation | March -April 2022  School A, monitored from 12/03 to 18/03, school B and C monitored from 19/03 to 25/03 and from 31/03 to 06/04 respectively | 3 naturally ventilated schools: 2 primary schools (A and B) and a secondary school (C). | Milan, Italy | The bigger the window opening ratio, the more the air change rates increase, the lower the probability of infection.  European recommendations for air renewal are achieved by a window opening area of at least 1.5 m.  The scenario with an infected teacher has slightly higher risk of infection compared to the scenario with an infected student | NATURAL VENTILATION | The Wells–Riley model assumptions: indoor air and aerosols are well mixed and the steady state (may not be held in larger rooms or those with irregular shapes and varies with ventilation rates). It also requires measurement of outdoor air supply rates |
|  | Associations between illness-related absences and ventilation and indoor PM_2.5_ in elementary schools of the Midwestern United States | Shihan Deng, Josephine Lau, Zhihao Wang , Pawel Wargocki | 2023 | Observational study:  Ventilation rates, indoor PM2.5 concentration, CO2 concentrations, student attendance records | To investigate the effects of indoor variables and absenteeism | 2 consecutive days every fall, winter, spring | 144 classrooms in 31 schools: 3105 students | Midwestern United States (Nebraska and Iowa), USA | Greater ventilation significantly reduced illness-related absences.  Higher concentrations of indoor PM_2.5_ were associated with increased illness-related absences.  Mechanically ventilated classrooms had relatively lower indoor PM_2.5_  Every 1 L/s per person increase in ventilation rate 🡪 5.59 decrease in absent days per year (0.15% increase in the annual daily attendance rate). Every additional 1 μg/m^3^ of indoor PM_2.5_ 🡪 7.37 increase in days with absences per year (0.19% decrease in the annual daily attendance rate).  Mean carbon dioxide concentrations were < 2,000 ppm  Mean indoor PM_2.5_ was 3.6 μg/m^3^ | MECHANICAL VENTILATION | -Discontinuous measures.  -Each classroom measured for 6 days |
|  | Window Opening or Mechanical Ventilation Systems for Italian Schools? Energetic Aspects, CO₂ Concentration and Infection Risk Assessment Based on the SARS-CoV-2 Data | Filippo Busato and Alberto Cavallini | 2023 | Model  IAQ, Wells-Riley model | To assess the effectiveness of natural ventilation and mechanical ventilation, with-without mask + assess infection risk  comparing the 2 different ventilation techniques (Wells-Riley equation) | 15 September to 15 June | School classrooms | Padova, Torino, Roma, Napoli, Palermo, Italy | Natural ventilation + Mask:  1 student is infected + after a 5 h class: the individual risk of infection is equivalent to 0.16 without a mask and 0.044 with it (4× risk reduction).  Mechanical ventilation + Mask:  forced ventilation system providing 40 m^3^/ (h person) of fresh outdoor air: infection probability decreases from 0.16 to 0.033 without a mask, and from 0.044 to 0.0083 with a mask.  Window opening was not efficient for preventing the risk of infection.  Mechanical ventilation reduced 3 times more the risk of infection, with and without a mask, and it could not only replace but even outperform the effect of masks.  With a full mask on, an infected teacher can still infect at least 1 student after a 2h class; without mechanical ventilation, an infected instructor could infect 3-6 children without a mask | NATURAL AND MECHANICAL VENTILATION  + MASK | The Wells–Riley model assumptions: indoor air and aerosols are well mixed and the steady state (may not be held in larger rooms or those with irregular shapes and varies with ventilation rates). It also requires measurement of outdoor air supply rates |
|  | Airborne infection risk in classrooms based on environment and occupant behavior measurement under COVID-19 epidemic | Lai Wei, Gang Liu, Wei Liu, Weifeng Li & Ye Yuan | 2023 | On site measurements: CO2 measurements, occupant area per person and activity level (survey) + Wells-Riley model to predict infection risk.  Observational study and mathematical modeling study | To assess  the infection risk in school classrooms  by including the environment and behavior  measurement  + calculate infection risk | 28 December  2020 to 8 January 2021 | 3 typical classrooms of a primary school | Tianjin, China | CO2 concentration 1000 ppm.  The infection risk transmission in the 3 classrooms was influenced by the concentration of people indoors, by the occupational area | AIR CLEANERS  + AIR PURIFIERS | Bias:  -distancing  -alternative model, proposed by Rudnick and Milton (2003), was adopted in this study to replace the ventilation rate by the rebreathed fraction calculated from indoor and outdoor CO2 concentration.  -age of students  -tightness and thermal insulation performance  -infection risk in this study is calculated under the  assumption of the complete mixing of air |
|  | The impact of retrofitted ventilation approaches on long-range airborne infection risk for lecture room environments: design stage methodology and application | Adam O’ Donovan, Paul D. O’ Sullivan | 2023 | Analytical multi-permutation static simulation approach (ventilation rate is obtained through infiltration and ventilation rate modelling based on established validated ventilation and infiltration models)  Mathematical modeling study | To evaluate different retrofitted ventilation approaches combined with additional control measures for evaluating the airborne infectious disease risk |  | Study lecture room (university classroom) | Dublin, Stockholm, Rome and Budapest (performance of all selected NV systems).  Provision of hybrid ventilation (HV) was assessed for the Dublin climate only | Mechanical ventilation system was found to be very effective at suppressing the virus infection risk.  Mask can suppress this risk substantially under the same external condition.  Natural ventilation is seasonally vulnerable | NATURAL AND MECHANICAL VENTILATION  +  MASK | Bias: face covering (masking) |
|  | A Monte Carlo Assessment of the Effect of Different Ventilation Strategies to Mitigate the COVID-19 Contagion Risk in Educational Buildings | Riccardo Albertin, Giovanni Pernigotto, and Andrea Gasparella | 2023 | Monte Carlo method + building performance simulation  Mathematical modeling study | To propose a new Monte Carlo method integrated with  building performance simulation to evaluate the number of infected occupants under different scenarios | Period 1, from 18.11.2018 to 23.12.2018, and period 2, from 08.11.2019 to 23.12.2019 | Study classroom at the Free University of  Bozen-Bolzano (24 students) | Bolzano, Italy | Importance of  combining different solutions (mixed-mode ventilation and masks) to limit the risk for both students and lecturers.  Natural ventilation can be useful to  further reduce the risk of contagion for those scenarios with a high number of susceptible subjects, where the combination  of mechanical ventilation and the open door fails to keep the  concentration level under an acceptable level | NATURAL AND MECHANICAL VENTILATION  Different scenarios:  (1)  Number of occupants (8, 16, or 24)  (2)  Type of control for mechanical ventilation (scheduled occupancy-based or CO_2_ concentration-based)  (3)  Status of the classroom door, closed or open (CO_2_ dilution factor equal to 1 or to 0.5) |  |
|  | Assessment of respiratory disease infection risk and natural ventilation intervention countermeasures in teaching spaces: A campus case study | Sui Li, Feifei Qin, Yixin Dong, Shiwen Zhou a, Jiayi Sun | 2023 | Mathematical model (Wells-Riley model and basic reproduction number R0 calculations) | To explore the interventions to prevent virus transmission, with a university campus |  | Shenyang Jianzhu University teaching space.  The classrooms in one unit have a capacity of 132 students (adults aged between 16 and 24) | Shenyang,northern China | Natural ventilation is not efficient to reduce the risk of infection.  With optimization:  indoor virus infection probability can be reduced by 22.88% | NATURAL AND MECHANICAL VENTILATION | -Number and activity of infected persons  -Intensity of activity of susceptible persons  -Setting of outdoor background meteorological parameters  -Activity trajectory of viral particles through the air and are similar to the particle distribution of microorganisms in the air |
|  | Ventilation strategy for simultaneous management of indoor particulate matter and airborne transmission risks-A case study for urban schools in South Korea | Junemo Koo, Young Min Jo, Tae Jeong Lee, Sowoo Park, Doosam Song | 2023 | CO2, PM, open/closed window and doors collection data.  Mathematical modeling study (Wells-Riley model) | To analyze the impact of ventilation rate on indoor PM2.5 concentrations and COVID-19 transmission risk in a typical Korean classroom | March-Nov 2021 | Classrooms of 13 schools in urban areas | South Korea | Quanta could be removed quickly with cross-ventilation with 100% of windows open. It is therefore recommended that classrooms be ventilated after school hours in full ventilation mode.  It was not possible to meet the safe condition without wearing a mask, and all 24 susceptible students were infected | NATURAL VENTILATION | -Typical dynamics of a Korean classroom  - The Wells–Riley model assumptions: indoor air and aerosols are well mixed and the steady state (may not be held in larger rooms or those with irregular shapes and varies with ventilation rates). It also requires measurement of outdoor air supply rates |
|  | Air Cleaners and Respiratory Infections in Schools: A Modeling Study Based on Epidemiologic, Environmental, and Molecular Data | Nicolas Banholzer, Philipp Jent, Pascal Bittel, Kathrin Zürcher, Lavinia Furrer, Simon Bertschinger, Ernest Weingartner,5 Alban Ramette, Matthias Egger, Tina Hascher, and Lukas Fenner | 2023 | Collection of environmental (CO_2_, particle concentrations), epidemiological (absences related to respiratory infections), audio (coughing), and molecular data (bioaerosol and saliva samples)  Observational study and mathematical modeling study | To examine the effectiveness of portable HEPA air filtration devices (air cleaners) in a school setting and the infection risk | 7 weeks during winter from Jan to March 2022/2023 | 2 Swiss secondary school classes (13*–*15 years).  In total 38 students | Solothurn, Switzerland | With air cleaners, mean particle concentration decreased by 77%. There were no differences in CO2 levels. Absences related to respiratory infections were 22 without air cleaners vs 13 with them.  The estimated number of respiratory infections in school would have been 19 if air cleaners had been installed throughout the study period, as compared with 36 if air cleaners had not been installed.  Coughing less frequent. Molecular analysis detected mainly non-SARS-CoV-2 viruses in saliva (50/448 positive), but not in bioaerosols (2/105 positive) or HEPA-filters (4/160) | AIR CLEANERS + HEPA FILTERS  Air cleaners improved air quality, showed a potential benefit in reducing respiratory infections. Associated with less coughing. Airborne detection of non-SARS-CoV-2 viruses was rare, suggesting that these viruses may be more difficult to detect in the air | -Aerosol measurements and molecular detection of viruses in bioaerosol samples document exposure (not transmission or direction)  -transmission route cannot be determined clearly: absences were self-reported by students, and some absences could have been incorrectly attributed to respiratory infections  -unmeasured confounding, (exposure outside of school)  -approximated incubation period for each epidemiologic case.  -not applicable to settings in the global south |
|  | Ventilation and thermal conditions in secondary schools in the Netherlands: Effects of COVID-19 pandemic control and prevention measures | Er Ding, Dadi Zhang, Amneh Hamida, Clara García-S´anchez, Lotte Jonker, Annemarijn R. de Boer, Patricia C.J.L. Bruijning, Kimberly J. Linde, Inge M. Wouters, Philomena M. Bluyssen | 2023 | Survey to monitor indoor and outdoor CO2 concentration and air temperature  + technical questionnaire and interview  Observational study | Monitoring indoor- outdoor CO2 concentration, air temperature, before and after the lockdown | October 2020 to June 2021, before and after a national lockdown (December 15, 2020 to March 1, 2021) | 31 classrooms in 11 Dutch secondary schools (age 12-18) | Netherlands | With windows and doors kept open: not achieved the ventilation standards.  Reducing occupancy can increase the ventilation rate per student in the classrooms | NATURAL AND MECHANICAL VENTILATION | - All the school visits were conducted during the heating season (situation during winter time non represented)  -Each school visit only lasted for 1 day (not all occupancies and behaviors in the classrooms were included)  -Steady-state condition  -Fixed number of occupants per lesson, of which the  -CO2 generation rate per person estimated as a fixed value for all occupants (no difference between sex, age, activity)  -No collection of occupants’ subjective assessments on the IEQ conditions of the classrooms |
|  | Assessment of natural ventilation strategy to decrease the risk of COVID 19 infection at a rural elementary school | Javier M. Rey-Hernandez, Yolanda Arroyo-Gomez, Julio F. San José Alonso Francisco J. Rey-Martínez | 2023 | Measurement of CO2, temperature, relative humidity, level of thermal comfort + decay CO2 concentration method +REHVA methodology (infection risks)  Observational study and mathematical modeling study | To evaluate the ventilation methods and the risk of infection transmission | Winter months of the COVID-19 pandemic | 9 classrooms of a public elementary school (Los Zumacales).  3 to 12 years old | Rural area of Castilla y Leon (North-Western Spain) | Only 1 classroom showed a value lower than 3 indicating poor ventilation.  The probability of infection in all the cases studied was less than 14%.  NECESSARY to open windows/doors at the end of each lecture | NATURAL VENTILATION | Bias:  Mandatory use of masks, capacity reduction, social distance, hand washing, disinfection of surfaces, isolation |
|  | Efficient trapping and destruction of SARS-CoV-2 using PECO-assisted Molekule air purifiers in the laboratory and real-world settings | Arpan Acharya, Kerri Surbaugh, Michellie Thurman, Chatura Wickramaratne, Philip Myers, Rajat Mittal, Kabita Pandey, Elizabeth Klug, Sarah J. Stein, Ashley R. Ravnholdt, Vicki L. Herrera, Danielle N. Rivera, Paul Williams, Joshua L. Santarpia, Ajeet Kaushik, Jaspreet S. Dhau, Siddappa N. Byrareddy | 2023 | Test chambe  Intervention study | To control the reduction of COVID-19 via nano-enabled filter technology using UV |  |  | University of Nebraska Medical Center (UNMC), USA | Efficient to remove Covid-19 from the air.  In 2 min, Molekule Air Mini+ removes 99.98 % of COVID-19 in an air chamber.  In 1 h, UV-A-stimulated PECO destructs over 99 % COVID-19 on filtration media.  Molekule Air-Pro air purifier removes COVID-19 from the air | AIR FILTRATION: PECO-assisted Molekule air purifiers |  |
|  | The effect of post-COVID-19 ventilation measures on indoor air quality in primary schools | Piet Eichholtz a, Nils Kok a,* and Xudong Sun | 2023 | Measurements of CO_2_ levels and fine particle concentration  Observational study | To evaluate ventilation rates and the effectiveness of building ventilation systems | September 2018 to July 2022 | 252 classrooms across 27 Dutch primary schools (5-12 yo) | Limburg province, Netherlands | Mechanically ventilated classrooms perform better with respect to CO2 and fine particle levels | NATURAL AND MECHANICAL VENTILATION |  |
|  | Evaluating the impact of air purifiers and window operation upon indoor air quality - UK nurseries during Covid-19 | Shuo Zhang, Samuel Stamp, Elizabeth Cooper, Katherine Curran, Dejan Mumovic | 2023 | Air quality sensor and passive sampling methods: PM2.5, volatile organic compounds, aldehydes  Observational study | To explore IAQ, the use of air purifiers and window operations | May 2028-2019 | 3 nurseries in London | London, UK | Mean reduction rate of PM2.5 by using air purifier was 63% with window closed, and 46% with window open.  Air purifiers can be effective at reducing PM2.5 when combined with proper window operation and have potential to bring substantial health benefits | NATURAL VENTILATION AND AIR PURIFIERS |  |
|  | The effect of natural ventilation on airborne transmission of the COVID-19 virus spread by sneezing in the classroom | Z.A. Firatoglu | 2023 | Experimental measurements for computational fluid dynamics (CFD) simulation result + effects of local flow behaviors on airborne transmission, for 5 scenarios using the Eulerian-Lagrange method, a discrete phase model, and a temporary three-dimensional CFD model.  Mathematical modeling study | To evaluate the effect  of natural ventilation on the airborne transmission of COVID-19-like | Jan-Feb, June | Secondary school classroom  (18 students) | Sanliurfa/Turkey | Natural ventilation in the classroom on the transmission of the virus travel was negligible | NATURAL VENTILATION |  |
|  | SARS-CoV-2 aerosol transmission in schools: the effectiveness of different interventions | Jennifer Villers, Andre Henriques, Serafina Calarco, Markus Rognlien, Nicolas Mounet, James Devine, Gabriella  Azzopardi, Philip Elson, Marco Andreini, Nicola Tarocco, Claudia Vassella, Olivia Keiser | 2022 | Aerosol transmission model  Mathematical modeling study | To study the effectiveness of natural ventilation, face masks, HEPA filtration  on the virus concentration |  |  |  | Combined interventions  (natural ventilation, masks, and HEPA filtration) were  the most effective.  HEPA filtration combined with face masks can reduce the mean aerosol concentration  by 90%.  The universal use of surgical masks was one of the most effective interventions | NATURAL AND MECHANICAL VENTILATION + HEPA + MASKS | -Lack of consensus regarding the infectious dose  -Assumption of a homogeneous viral concentration  -No account for the proximity to the infectious individual |
|  | Quantitative Assessment of Natural Ventilation in an Elementary School Classroom in the Context of COVID-19 and Its Impact in Airborne Transmission | Andrés Vignolo, Ana Paula Gómez, Martín Draper and Mariana Mendina | 2022 | CO2 measurements (5 months) + ventilation rates + infection risk calculations (Wells-Riley) + analytical simulations for different  scenarios  Observational study and mathematical modeling study | To calculate CO2 concentration for 5 months and assess the effectiveness of different interventions on the reduction of COVID-19 transmission risk | 5 months in 2021 | 2 elementary schools in Uruguay (1 class= 30 children + 1 teacher) | Uruguay | Periodic ventilation is an extremely efficient strategy  for reducing transmission risk.  Ventilation rates improved from winter to spring, (decline in estimated infection risk) | NATURAL VENTILATION | -Variable duration of windows ‘adjustment (high ACH results could inflate results  -Perfect mix hypothesis  -Rudnick-Milton approach limitations: using CO2 as a proxy but COVID-19 virus is transmitted via small droplets of fluid. The movement and dispersion are more complex than that of gaseous CO2 and is highly dependent on the droplet size |
|  | Monitoring CO₂ concentration to control the infection probability due to airborne transmission in naturally ventilated university classrooms | Fabio Fantozzi, Giulia Lamberti, Francesco Leccese & Giacomo Salvadori | 2022 | CO2 measurements, Wells-Riley equation for infection risk.  Observational study and mathematical modeling study | To estimate the probability of infection from CO2 concentration  monitoring | December 2018 | 20 university classrooms | Pisa, Italy | Elevated CO₂ levels correlate with high risk of airborne transmission of COVID-19.  Higher class occupancy and increased activity levels lead to elevated CO₂ concentrations.  EFFECT OF MASKS :  effective FFP2 mask is the only measure that allows for keeping  the infection probability lower than 1%.  Cloth or surgical masks are  not sufficient to keep the infection probability below 1% for one  hour of exposure, while highly effective masks are efficient in  all the configurations | NATURAL VENTILATION | -Ventilation rate assumed to be constant  -Quantum generation rate produced by one infector is not known (assumed constant and equal  to values provided by medical studies)  -No account for different susceptibilities among non-infected individuals  -Viral exposure concentration  was considered uniform across the room (no higher close to the infector) |
|  | Modeling impacts of ventilation and filtration methods on energy use and airborne disease transmission in classrooms | Theresa Pistochini, Caton Mande, Subhrajit Chakraborty | 2022 | Simulation of outdoor air ventilation rates and filtration methods, modified Wells-Riley equation and EnergyPlus building simulation  Mathematical modeling study | To understand the trade-offs between infection probability and energy consumption | School year: from the third Monday in August and lasts for 36 weeks | Classroom in 13 large cities: Miami, Houston, Phoenix, Atlanta, Los Angeles, San Francisco, Las Vegas, Baltimore, Albuquerque, Seattle, Chicago. Denver, Minneapolis | USA | Infection probability was 29% lower with improved filtration.  (either though use of MERV 13 filters, IRF, or both) in comparison to increasing ventilation rates above 7.0 l/(person) | HVAC filtration system model | The Wells–Riley model assumptions: indoor air and aerosols are well mixed and the steady state (may not be held in larger rooms or those with irregular shapes and varies with ventilation rates). It also requires measurement of outdoor air supply rates |
|  | Long-term filter efficiency of mobile air purifiers in schools | Manuel Granzin, Sarah Richter, Jann Schrod, Natalie Schubert & Joachim  Curtius | 2022 | Monitoring of devices and particles decay  Observational study | To evaluate air purifiers  with HEPA filters  + the efficiency and long-term performance of 3 devices over 6  month by monitoring the particle decay from 0.003 to 10 mm | November 2020- May 2021 | 2 rooms: seminar room at Goethe University  Frankfurt, + high  school Kaiserin-Friedrich-Gymnasium (KFG) (students from grades 5 to 12, age from  10 to 19, in Bad Homburg  vor der Hohe, Germany  (minimum of 8 students  up to 28 students plus one teacher) | Germany | With 1 infected person in the classroom:  the additional use of air purifiers reduced the number of inhaled particles viruses at the end of the day by a factor of 2.65, compared to without air purifiers | HEPA FILTERS |  |
|  | Assessment of ventilation rates inside educational buildings in Southwestern Europe: Analysis of implemented strategic measures | Antonio J. Aguilar, María L. de la Hoz-Torres, Nélson Costa, Pedro Arezes, María D. Martínez-Aires , Diego P. Ruiz | 2022 | In situ measurements, ventilation rate using the CO_2_ decay method, probability of infection risk using the Wells-Riley equation  Observational study | To assess ventilation rate and ventilation strategies | March-July 2021 | 14 classrooms: 8 on Azurém Campus (Guimaraes, Portugal) and 6 on the Fuentenueva Campus (Granada, Spain) | Portugal, Spain | Ventilation rates: between 2.9 and 20.1 air change per hour (ACH) for natural cross ventilation, 2.0 to 5.1 ACH for single-sided ventilation and 1.8 to 3.5 for mechanically ventilated classrooms.  The probability of risk was less than 1% in almost all of the classrooms analyzed.  Natural ventilation (door and windows opening) can provide sufficient air renewal maintaining a low risk of infection | NATURAL + MECHANICAL VENTILATION | The Wells–Riley model assumptions: indoor air and aerosols are well mixed and the steady state (may not be held in larger rooms or those with irregular shapes and varies with ventilation rates). It also requires measurement of outdoor air supply rates |
|  | Assessment of indoor air quality and risk of COVID-19 infection in Spanish secondary school and university classrooms | Diana Rodríguez, Itziar R. Urbieta, Angel Velasco, Miguel Angel Campano-Laborda, Elena Jiménez | 2022 | Outdoor- indoor CO_2_ levels, relative transmission risk of COVID-19 using the web app *COVID Risk^airborne^*.  Observational study | To evaluate  the ventilation conditions, COVID-19 risk, and IAQ | Nov 2020-June 2021 | Secondary school and University in Toledo, Spain | Toledo, Spain | Mechanical ventilation: better ventilation conditions + maintaining an acceptable risk of transmission, especially when the use of masks was considered | NATURAL + MECHANICAL VE TILATION | Bias: use of mask |
|  | Increasing ventilation reduces SARS-CoV-2 airborne transmission in schools: a retrospective cohort study in Italy’s Marche region | Giorgio Buonanno, Luca Ricolfi, Lidia Morawska and  Luca Stabile | 2022 | Retrospective cohort study  Intervention study | To assess the association between ventilation  and COVID-19 transmission | 13 September 2021 and 31  January 2022 | 1419 schools (10441 classrooms in in total): 56 schools (316 classrooms) equipped with mechanical ventilation (single room ventilation units, filters and heat recovery).  Average occupancy of 20 students  per classroom (total of 205247 students) | Marche region, Italy | Mechanical ventilation systems to protect  students in classrooms from airborne transmission, lower risk compared to natural ventilation.  For classrooms equipped with mechanical ventilation systems, the relative risk of infection of students decreased at least by 74% compared with a classroom with only natural ventilation, reaching values of at least 80% for ventilation rates >10 L s^-1^ student^-1^.  Classes with mechanical ventilation: 31 infected students.  With natural ventilation: 3090 infected students | NATURAL + MECHANICAL VENTILATION | Bias of additional measures: masks, distancing |
|  | ENERGY PERFORMANCE AND INFECTION RISK EVALUATION OF RETROFITTED VENTILATION SYSTEMS IN TIMES OF COVID | Fatos Pollozhani, Robert S. McLeod, Thomas Klimach,  Ulrich Pöschl Christina J. Hopfe | 2022 | Simulation model  Mathematical modeling study | To evaluate the viral transmission patterns and energetic performance of natural and mechanical ventilation compared to MPIC-MEV systems | All year round.  For COVID19 risk assessment: summer conditions simulation (30^th^ June) | Lecture room | Ireland | COVID19 risk assessment under summer conditions (30^th^ June).  CO2:  Scenario 1: >1000 ppm during an 8-hour period without active ventilation. Scenarios 2 and 3: mechanically driven by fans, the indoor CO2 concentrations 950 ppm over the whole year.  Scenario 3: decrease in indoor CO2 concentration during lunch break compared to scenario 2.  Scenario 5: values ranging from 600 to 1600 ppm. because of the purge-vent cycles.  COVID19 risk:  1 person infected with a class n=20, n=30.  Scenario 1: highest individual risk: 17% (no active ventilation). With n=30, 100% combined risk.  Scenario 2: the lowest risk: 2%. With n=30: 49% combined risk.  Scenario 3: individual risk of 3%. With n=30: 59%. Due to reduced emission intensity caused by the distributed extraction of aerosols by the MPIC-MEV system extract hoods.  Scenario 4: individual risk of 4% and combined risk 74% if n=30.  Scenario 5: individual infection risk of 3%, combined risk of 61% with n=30.  Scenario 2 performs well for IAQ, maintaining constant CO2 values over the whole year + lower infection risk in summer.  The retrofitted mechanical ventilation system significantly enhanced indoor air quality compared to natural ventilation strategies and decreased risk transmission of COVID-19 | NATURAL, MECHANICAL AND ALTERNATIVE VENTILATION (MPIC-MEV systems)  No use of face masks  5 different scenarios:  Scenario 1 infiltration and exfiltration but no active ventilation (base case). 2 (MPIC MEV) and 3 scenario: mechanical ventilation.  Scenario 4: natural ventilation with tilted windows and 5 natural ventilation with purge ventilation | -Buoyancy effects were not quantified  -Exclusion of the room air displacement effects |
|  | Airborne infection risks of SARS-CoV-2 in US schools and impacts of different intervention strategies | Yifang XU, Jiannan CAI, Ph.D., Shuai LI, Ph.D., Qiang HE, Ph.D., Siyao ZHU | 2021 | Scenario-based analysis + epidemiological scenarios  + infection risk modeling  Mathematical modeling study | To estimate the infection risk of COVID-19 in 111485 schools and to evaluate the impacts of different intervention strategies (increased ventilation, air filtration, hybrid learning) | 1 year period with pandemic scenario | 111 485 public and private schools: 90,160 public and 21,325 private | USA | Infection risk in 90% of the schools: >1%. Lower in July (3.85%) compared to December (6.83%).  Infection risk decreases by 16.5% by increasing ventilation rate from 2/h to 2.5/h and by 8% from 5.5/h to 6/h.  Air filtration is found to be most effective: the school average infection risk when applying MERV 13 is over 30% less than the risk levels correlating with the use of increased ventilation and hybrid learning.  It is necessary to combine intervention strategies for infection risk< 1%: further increasing the ventilation rate, use of filters with a higher MERV rating, and adopting online learning (reduction of occupancy) | AIR FILTRATION |  |
|  | Mitigating aerosol infection risk in school buildings: the role of natural ventilation, volume, occupancy and CO_2_ monitoring | Alessandro Zivelonghi, Massimo Lai | 2021 | CO_2_ measurements with real occupants +comprehensive risk mitigation strategy  Mathematical modeling study and observation | To assess a quantitative analysis based on the GN-Riley infection risk model | June 2021 | 1 high school (1 classroom) | North of Italy | The regular windows opening at defined intervals is not sufficient. It is necessary to combine it with other measures:  microphone (20% risk reduction (without masks) almost 40% with surgical masks, and increasing the classroom size (risk reduced by half) | NATURAL (during breaks) AND MECHANICAL VENTILATION  + model with-without masks and microphone for the teacher |  |
|  | Natural ventilation strategy and related issues to prevent coronavirus disease 2019 (COVID-19) airborne transmission in a school building | Sowoo Park , Younhee Choi, Doosam Song, Eun Kyung Kim | 2021 | Measurement of CO_2_ decay by cross vs. single-sided ventilation with 0%, 15%, 30% and 100% window opening ratio (when unoccupied classroom) + Wells-Riley model (different scenarios with 0.5 h to 3 h exposure time)  Observational study mathematical modeling study | To analyze window opening rates to prevent viral air infection | May 2020 | 1 elementary school (3 classrooms) | South Korea | Natural ventilation not enough: the infection probability can be reduced by about 4 times when a mask was worn under the same ventilation rate conditions  + better cross ventilation compared to single sided ventilation.  Without ventilation: infection risk >1% even with mask and exposure time of 0.5h.  Infection risk <1% with cross ventilation without mask with 30% window opening and 1 h exposure. Infection risk of <1% with single sided ventilation only with masks and exposure time of max. 1 h | NATURAL VENTILATION: cross-sided and single sided ventilation  . | Wells-Riley equation assumes well mixed air and steady state infection particle concentration which varies with ventilation rates |
|  | Implementation of a Ventilation Protocol for SARS-CoV-2 in a Higher Educational Centre | Alberto Meiss, Irene Poza-Casado, Alfredo Llorente-Álvarez, Héctor Jimeno-Merino  and Miguel Ángel Padilla-Marcos | 2021 | Experimental study in a typical classroom  Intervention study |  | 2020-2021 school year | University center | Valladolid (Spain) | The combined action of natural ventilation, air conditioning system (with 50% of outdoor air) and filtering device is the strategy that removes particles and aerosols  in the shortest time | NATURAL VENTILATION + MECHANICAL and AIR PURIFIERS |  |
|  | Ventilation procedures to minimize the airborne transmission of viruses in classrooms | L. Stabile, A. Pacitto, A. Mikszewski, L. Morawska, G. Buonanno | 2021 | Simulation of 5h school day  Mathematical modeling study |  |  | High-school classroom (students aged 17–18) (25 pp in the room) |  | Air exchange rate <0.1/h for preventing seasonal influenza.  9.5/h and 0.8/h if the teacher is infector to prevent a COVID-19 infection (60 min speaking versus through microphone). Required AER if the student is infector depends on speaking time and attendance: 0.8–3.5/h.  Long ventilation periods or high air exchange rates not always possible with natural ventilation.  Mitigation solutions (voice modulation) or immunization are needed | NATURAL AND MECHANICAL VENTILATION: simulation of different scenarios (different infectors, intensity of speaking) |  |
|  | Indoor Air Quality in Naturally Ventilated Classrooms. Lessons Learned from a Case Study in a COVID-19 Scenario | Alberto Meiss, Héctor Jimeno-Merino, Irene Poza-Casado, Alfredo Llorente-Álvarez  and Miguel Ángel Padilla-Marcos | 2021 | Air quality and hygrothermal comfort conditions before and after the pandemic  Observational study | To evaluate the efficiency of different implementation of ventilation strategies | September 2020 | Nursery and primary public school | Valladolid (Castilla y León, Spain | Mixing model, which ensures the removal of pollutants, is guaranteed by continuous cross-ventilation | Different ventilation scenarios |  |
|  | CO₂ concentration monitoring inside educational buildings as a strategic tool to reduce the risk of Sars-CoV-2 airborne transmission | Alessia Di Gilio, Jolanda Palmisani, Manuela Pulimeno, Fabio Cerino, Mirko Cacace, Alessandro Miani, Gianluigi de Gennaro | 2021 | CO2 surveillance activity for 3 weeks | Pilot study  Observational study | 18th January to February 8, 202 | 9 schools: 11 classrooms: 2 pre-school (3–6 years old children), 5 primary (6–11 years old pupils) and 4 first-level secondary classrooms (11–13 years old students) | Apulia Region (South of Italy | Only natural ventilation was not effective to guarantee adequate air exchanges🡪further strategies based on heating, ventilation, air conditioning systems, air cleaner.  Indoor CO_2_ monitoring is a practical tool for COVID-19 transmission risk.  All classrooms showed inadequate ventilation conditions (CO_2_ higher than 700 ppm) | NATURAL VENTILATION:  Opening-closing windows and doors not scheduled |  |
|  | Study on Droplet Dispersion Influenced by Ventilation and Source Configuration in Classroom Settings Using Low-cost Sensor Network | Weiqi Chen, Dong-Bin Kwak, Jonathon Anderson, Kaushik Kanna, Chenxing Pei, Qingfeng Cao, Qisheng Ou*, Seong Chan Kim, Thomas H. Kuehn, David Y.H. Pui | 2021 | Monitoring of airborne particle concentration + simulation  Observational study and mathematical modeling study | To develop a low-cost wireless particle concentration monitoring network (PCMN) and to study the respiratory droplet dispersion |  | University classroom: 2 types: air handling unit (AHU) and fan coil unit (FCU) | University of Minnesota, USA | 2 recommendations on reducing the viral risk:  1.the respiratory droplet source should be in the location such that the particle dispersion opposes the ventilation flow.  2.the air handling unit (AHU) and fan coil unit (FCU) should be both turned on during class hours | MECHANICAL VENTILATION |  |
| Mask Use and Ventilation Improvements to Reduce COVID-19 Incidence in Elementary Schools — Georgia | | Jenna Gettings, DVM; Michaila Czarnik, MPH; Elana Morris, MPH; Elizabeth Haller, MEd; Angela M. Thompson-Paul, PhD; Catherine Rasberry, PhD; Tatiana M. Lanzieri, MD; Jennifer Smith-Grant, MSPH; Tiffiany Michelle Aholou, PhD; Ebony Thomas, MPH; Cherie Drenzek, DVM; Duncan MacKellar, DrPH | 2021 | Survey in schools (on prevention strategies: increased natural ventilation, filtration, masks, physical distancing, desks barriers, cohort size) + mathematical modeling study | To evaluate the impact of preventive strategies against COVID-19 among students, without COVID-vaccines | Nov 16-Dec 11 2020 | Kindergarten-grade 5 (1321 schools) and 140 private schools.  169 (11.6%) of 1,461 schools in 51 (32.1%) of 159 Georgia counties (median = two schools per county) completed the survey and also had available COVID-19 case data | Georgia, USA | 3.08 cases among students and teachers per 500 students enrolled.  The incidence of COVID-19 was 37% lower in schools where teachers used masks.  21% lower in schools where students had a mask.  39% lower in schools with improved ventilation.  Ventilation strategies: opening windows, doors, fans (35% lower incidence).  HEPA with/ without purification UVGI (48% lower incidence).  MULTIPLE STRATEGIES ARE SUGGESTED (ventilation improvements + masks for teachers) | MASKS +  VENTILATION  Preventive strategies:  -Masks (teachers, staff 65.1% of the schools), students (51.5% of the schools))  -Ventilation strategies (51.5%)  -Physical distance (18.9%)  -Barriers on desks (22.5%)  -Small classes  -Handwash  -Medical leave for teachers (81.7%) | -Bias of preventive measures  -Self-reported cases of COVID-19  -Limited power for air filtration and purification (only 16 schools)  -Response rate of the survey was low (11.6%).  -Missing info for ventilation strategies.  -No causal relationships |
|  | The Impact of Large Mobile Air Purifiers on Aerosol Concentration in Classrooms and the Reduction of Airborne Transmission of SARS-CoV-2 | Finn F. Duill , Florian Schulz, Aman Jain, Leve Krieger, Berend van Wachem and Frank Beyrau | 2021 | Measurements of the number of particles, the particle size distribution, and the CO_2_ concentration  Observational study | To evaluate the effect of large mobile air purifiers (AP) with HEPA filters | April-May 2021 | Primary school: 3 classrooms | Germany | Room size, duration of stay, and activity of the people in the  Room: major influence on the inhaled dose of virus-laden particles.  The use of air purifier: positive effect in reducing virus-laden aerosols  when people are in small rooms for long periods.  Window ventilation reduces respiratory particle concentration and improves air  quality in terms of humidity and CO2, but it is not reliable  to reduce the concentration of virus-laden particles | AIR PURIFIER with HEPA filters VS NATURAL VENTILATION.  Natural ventilation for 5 minutes every 20 minutes during lessons vs no ventilation | Bias: reduced occupancy |
|  | High energy efficiency ventilation to limit COVID-19 contagion in school environments | Luigi Schibuola, Chiara Tambani | 2021 | Measurements of indoor/outdoor CO_2_ concentrations to calculate actual air changes per hour  Observational study and mathematical model | To evaluate the current infectious risk | Winter 2015-2016 | 2 secondary schools: 4 classrooms | Treviso and Cremona, Italy | With natural ventilation, frequent and short periods of ventilation reduce CO2 concentration, but the recommended concentration is not guaranteed all the time. Dangerous in the presence of an asymptomatic person.  Infection risk >1%.  Mechanical ventilation reduced risk from 23% (8 L s^−1^ person^−1^) to 7.2% (32 L s^−1^ person^−1^) with additional filtration (efficiency 95%): 0.38%.  Reduction  of indoor viral concentration and consequently of the infection risk by a strong mechanical ventilation. High ventilation rates permit limited facemask use to have R0 <1 | NATURAL VENTILATION with different ventilation scenarios + MECHANICAL VENTILATION with different ACH and normal occupancy |  |
|  | Seasonal variation in airborne infection risk in schools due to changes in ventilation inferred from monitored carbon dioxide | Carolanne V. M. Vouriot,\| Henry C. Burridge Catherine J. Noakes Paul F. Linden | 2021 | CO2 data + risk modeling based on Wells-Riley model for 5 days in January and July 2018  Observational study + mathematical modeling study | To investigate the infection disease transmission in school environment. | November 2015 to March 2020 | 45 classrooms in 11 schools (8 primary, 3 secondary) | UK | January is twice as risky as July for COVID-19 transmission, due to changes in ventilation rates.  CO2:  decrease in CO2 levels, both in terms of daily mean and of peak values, in July compared to January.  Classrooms with more active-vocal activities:  the airborne risk of transmission increases  by a factor of nearly 5 | HYBRID: NATURAL + MECHANICAL VENTILATION | The Wells–Riley model assumptions: indoor air and aerosols are well mixed and the steady state (may not be held in larger rooms or those with irregular shapes and varies with ventilation rates). It also requires measurement of outdoor air supply rates |
|  | Assessment of CO_2_ and aerosol (PM_2.5_, PM_10_, UFP) concentrations during the reopening of schools in the COVID-19 pandemic: The case of a metropolitan area in Central-Southern Spain | Florentina Villanueva, Alberto Notario, Beatriz Cabañas, Pilar Martín, Sagrario Salgado, Marta Fonseca  Gabriel | 2021 | IEQ monitoring + survey  Observational study |  | September 30th until October 27th 2020 | 7 schools, 19 classrooms (6 pre-schools, 7 primary and 6 secondary) | Ciudad Real, Central-Southern Spain | 5 (26%) surveyed classrooms had CO2 concentration values >700 ppm.  Preschool rooms had better ventilation, while secondary classrooms showed the highest peak and average CO2 concentrations. For PM2.5, PM10 and UFP, in preschools were 2-fold greater than the levels obtained in both primary and secondary classrooms.  CROSS VENTILATION: does not seem to be enough for providing adequate ventilation rate, at the existing occupancy | CROSS NATURAL VENTILATION: all windows and doors open.  Only 1 classroom was MECHANICALLY VENTILATED | Bias:  hand hygiene, cleaning of surfaces, maximizing physical distance to maintain at least 1.5 m distance (whenever possible) + the use of face masks has been mandatory for the whole period while at school, indoors and outdoors, except for preschool children |
|  | Effects of the COVID-19 Pandemic on Indoor Air Quality and Thermal Comfort of Primary Schools in Winter in a Mediterranean Climate | Alicia Alonso,  Jesús Llanos,  Rocío Escandón   and  Juan J. Sendra | 2021 | Onsite measurements conducted before and during the pandemic: air temperature, relative humidity, and level of CO₂ concentration.  Observational study | To analyze the effects of COVID-19 pandemic  on thermal comfort and indoor air quality | 13 weeks in winter before and during the pandemic: From 23 January to 13 March 2020, and From 30 November 2020 to 22 January 2021 | 2 classrooms f 1 elementary school | Sevilla, Spain | Decrease of 300 ppm in CO2 weekly average values during the  pandemic, when hybrid ventilation is used, and a decrease of 400 ppm when natural ventilation is added.  Mean CO_2_ conc. 1033 ppm or 1079 ppm in both classrooms with only mechanical ventilation. CO_2_ conc. 604 ppm or 740 ppm when additional natural ventilation is used | MECHANICAL VENTILATION and NATURAL VENTILATION  Mechanical ventilation measurements of CO2. Sometimes additional natural ventilation.  Both classrooms have a mechanical ventilation but  only A5 can provide cross-ventilation while A6 only provides ventilation from  one side |  |
|  | Testing mobile air purifiers in a school classroom: Reducing the airborne transmission risk for SARS-CoV-2 | [J. Curtius](https://www.tandfonline.com/author/Curtius%2C+J),  [M. Granzin](https://www.tandfonline.com/author/Granzin%2C+M),  [J. Schrod](https://www.tandfonline.com/author/Schrod%2C+J) | 2021 | Observational study: IAQ measurements without purifiers(only with natural ventilation), plus 3 or 4 air purifiers(natural ventilation: up to 5 windows open + door) | To test the effectiveness of mobile air purifiers to reduce the transmission risk of COVID-19 | Heating period before and during the COVID-19 pandemic | 1 school (2 high school classrooms): 27 students per class | Germany | Air purifiers are an effective measure to reduce the transmission risk of COVID-19:  With all windows and door closed within the intervals between venting by opening windows and door, the aerosol concentration was reduced by more than 90% in less than 30 min with the air purifier | 4 AIR PURIFIERS with HEPA filters vs NATURAL VENTILATION |  |
|  | Estimating aerosol transmission risk of SARS-CoV-2 in New York City public schools during reopening | Brian Pavilonis, A. Michael Ierardi,LeonLevine,Franklin Mirer,Elizabeth A. Kelvin | 2021 | IAQ survey (CO2, temperature, relative humidity) + Wells-Riley equation  Observational study and mathematical modeling study | To estimate the risk of transmission of COVID-19 | December 2017-Spetember 2018 | 19 elementary schools (101 classrooms: 86 during heating season, 69 during cooling season, 54 in both).  Range: 9-30 students | New York, USA | Risk transmission rates were 28% greater in the heating compared to cooling season, in schools in higher income area schools and in new buildings.  It was lower in schools with mechanical ventilation.  Mask wearing was associated with lower transmission rate, which was higher from an infected teacher to student compared to infected student to teacher or student to student | NATURAL VENTILATION + MECHANICAL VENTILATION (37% of schools) | The Wells–Riley model assumptions: indoor air and aerosols are well mixed and the steady state (may not be held in larger rooms or those with irregular shapes and varies with ventilation rates). It also requires measurement of outdoor air supply rates |
|  | A systematic approach to estimating the effectiveness of multi-scale IAQ strategies for reducing the risk of airborne infection of SARS-CoV-2 | Jialei Shen, Meng Kong, Bing Dong, Michael J.Birnkrant, Jianshun Zhang | 2021 | Infection risk estimation model: Wells-Riley.  Mathematical modeling study | To analyze the effectiveness of different strategies to reduce COVID-19 airborne transmission |  | Different indoor spaces: K-12 schools |  | Higher probability of COVID-19 transmission from teacher to students (13.2%) than student to student (3.8%).  Reduction of infection risk: 37% reduction when doubling total supply airflow rate.  100% reduction with outdoor air.  27% reduction with HEPA filter.  26% reduction when displacement ventilation.  46% reduction when partitions.  46% reduction with personal ventilation | NATURAL VENTILATION, HEPA FILTERS | The Wells–Riley model assumptions: indoor air and aerosols are well mixed and the steady state (may not be held in larger rooms or those with irregular shapes and varies with ventilation rates). It also requires measurement of outdoor air supply rates |
|  | Estimating the nationwide transmission risk of measles in US schools and impacts of vaccination and supplemental infection control strategies | [Parham Azimi](https://pubmed.ncbi.nlm.nih.gov/?size=50&term=Azimi+P&cauthor_id=32652940), [Zahra Keshavarz](https://pubmed.ncbi.nlm.nih.gov/?size=50&term=Keshavarz+Z&cauthor_id=32652940), [Jose Guillermo Cedeno Laurent](https://pubmed.ncbi.nlm.nih.gov/?size=50&term=Cedeno+Laurent+JG&cauthor_id=32652940), [Joseph G Allen](https://pubmed.ncbi.nlm.nih.gov/?size=50&term=Allen+JG&cauthor_id=32652940) | 2020 | Wells-Riley approach, nationwide representative School Building Archetype (SBA) model, and Monte-Carlo simulation.  Estimates were validated with real outbreak data.  Mathematical modeling study | To evaluate the effect of vaccination, compartmentalizing and ventilation/purification/filtration on measles transmission |  | Primary and secondary schools | USA | Transmission risk 74 times higher for unvaccinated students, higher in high schools (median 5.8%) than in elementary schools (median 3.8%).  Highest transmission risk (median 6%) in schools with ductless systems without air filters.  Lowest transmission risk (3.7% median) in schools with ductless systems with air filters.  For unvaccinated students: using a better filter reduced transmission risk (median 45% for MERV8, 32% for MERV13, and 29% for HEPA filter), and also increasing ventilation rates (46% basic control, 38% regular, 33% advanced infection scenario) | Ventilation/purification/filtration (MERV, HEPA) | The Wells–Riley model assumptions: indoor air and aerosols are well mixed and the steady state (may not be held in larger rooms or those with irregular shapes and varies with ventilation rates). It also requires measurement of outdoor air supply rates |

| Improving indoor air quality in schools: Evidence from an air purifier intervention with low-cost sensors  PRE-PRINT | Stefania Renna, Jacopo Bonan, Francesco Granella, and Luis Sarmiento | 2025 | RCT | To measure the cost-effectiveness of portable air purification systems in classrooms. |  | 5 primary schools: 95 classrooms (2000 students) (treatment and control groups randomly assigned) | Milan, Italy | Air purifiers reduced air pollution in classrooms by 28% + 11% of student absenteeism.  The effect was greater in vulnerable and non-Italian students.  Limitations to maintain good indoor air quality when outdoor air is very polluted. | AIR PURIFIERS |  |
| --- | --- | --- | --- | --- | --- | --- | --- | --- | --- | --- |
| EMPA study  Not published |  |  | 300 CO2 sensors + weekly COVID-19 tests | To measure the correlation between poor ventilation and COVID-19 incidence | 1-19 Nov 2021 | 150 classrooms of 59 primary schools | Graubünden | Significant correlation between poor ventilation and COVID-19 cases.  60% of the classrooms: CO2 levels >2000 ppm |  |  |

| **Title** | **Author(s) names** | **Date of publication** | **Study design** | **Study period of investigation** | **Type of indoor setting** | **Main results** | **Articles** |
| --- | --- | --- | --- | --- | --- | --- | --- |
| Ventilation in school and students' health after outbreak of COVID-19: A systematic literature review | Moelyaningrum AD, Keman S, Melaniani S, Prasasti CI. | 2024 | Literature Review | 2019-May 2023 | Primary, middle and secondary school | Diseases identified in students as a result of poor ventilation: tiredness, bronchitis, asthma, infection risk, rhinitis. Ventilation reduces indoor air pollutants and it important is to fulfil the requirements for student’s health | 1. Callies, M., Kabouche, I., Desombere, I., Merckx, J., Roelants, M., Vermeulen, M., & Duysburgh, E. (2023). SARS-CoV-2 infection prevention and control measures in Belgian schools between December 2020 and June 2021 and their association with seroprevalence: a cross-sectional analysis of a prospective cohort study. *BMC public health*, *23*(1), 898. <https://doi.org/10.1186/s12889-023-15806-5> 2. Cordery, R., Reeves, L., Zhou, J., Rowan, A., Watber, P., Rosadas, C., Crone, M., Storch, M., Freemont, P., Mosscrop, L., Cowley, A., Zelent, G., Bisset, K., Le Blond, H., Regmi, S., Buckingham, C., Junaideen, R., Abdulla, N., Eliahoo, J., Mindlin, M., … Sriskandan, S. (2022). Transmission of SARS-CoV-2 by children to contacts in schools and households: a prospective cohort and environmental sampling study in London. *The Lancet. Microbe*, *3*(11), e814–e823. https://doi.org/10.1016/S2666-5247(22)00124-0 |
| Indoor Air Quality and COVID-19: A Scoping Review | Axelle Braggion, Adeline Dugerdil, Olwen Wilson, Francesca Hovagemyan and Antoine Flahault | 2024 | Scoping review | January 2020-October 2022 | Workplaces, schools, restaurants, and public transport | Indoor air quality can be improved with ventilation strategies. This could reduce the risk of developing COVID-19 | 1. Gettings, J., Czarnik, M., Morris, E., Haller, E., Thompson-Paul, A. M., Rasberry, C., Lanzieri, T. M., Smith-Grant, J., Aholou, T. M., Thomas, E., Drenzek, C., & MacKellar, D. (2021). Mask Use and Ventilation Improvements to Reduce COVID-19 Incidence in Elementary Schools - Georgia, November 16-December 11, 2020. *MMWR. Morbidity and mortality weekly report*, *70*(21), 779–784. https://doi.org/10.15585/mmwr.mm7021e1 2. Lasser, J., Sorger, J., Richter, L., Thurner, S., Schmid, D., & Klimek, P. (2022). Assessing the impact of SARS-CoV-2 prevention measures in Austrian schools using agent-based simulations and cluster tracing data. *Nature communications*, *13*(1), 554. https://doi.org/10.1038/s41467-022-28170-6 |
| Assessing Indoor Air Quality and Ventilation to Limit Aerosol Dispersion-Literature Review | Nadine Hobeika, Clara García-Sánchez and Philomena M. Bluyssen | 2023 | Literature review | 2013-2023 | Schools or offices | To limit aerosol dispersion indoors, there is a need for better coordination between different fields | 1. Zivelonghi, A., & Lai, M. (2021). Mitigating aerosol infection risk in school buildings: The role of natural ventilation, volume, occupancy and CO2 monitoring. *Building and Environment*, *204*, 108139. <https://doi.org/10.1016/j.buildenv.2021.108139> 2. Hou, D., Katal, A., & Wang, L. (2021). Bayesian Calibration of Using CO2 Sensors to Assess Ventilation Conditions and Associated COVID-19 Airborne Aerosol Transmission Risk in Schools. *medRxiv*.Ascione F. et al (2021) 3. Ascione, F., De Masi, R. F., Mastellone, M., & Vanoli, G. P. (2021). The design of safe classrooms of educational buildings for facing contagions and transmission of diseases: A novel approach combining audits, calibrated energy models, building performance (BPS) and computational fluid dynamic (CFD) simulations. *Energy and Buildings*, *230*, 110533. <https://doi.org/10.1016/j.enbuild.2020.110533> 4. Park, S., Choi, Y., Song, D., & Kim, E. K. (2021). Natural ventilation strategy and related issues to prevent coronavirus disease 2019 (COVID-19) airborne transmission in a school building. *The Science of the total environment*, *789*, 147764. <https://doi.org/10.1016/j.scitotenv.2021.147764> 5. Foster, A., & Kinzel, M. (2021). Estimating COVID-19 exposure in a classroom setting: A comparison between mathematical and numerical models. *Physics of fluids (Woodbury, N.Y. : 1994)*, *33*(2), 021904. https://doi.org/10.1063/5.0040755 |
| The Influence of Ventilation Measures on the Airborne Risk of Infection in Schools: A Scoping Review | Sandra N. Jendrossek, Lukas A. Jurk, Kirsten Remmers, Yunus E. Cetin, Wolfgang Sunder,  Martin Kriegel and Petra Gastmeier | 2023 | Scoping Review | Before 1 May 2022 | Schools | The ventilation in many schools is not adequate to guarantee good indoor air quality. Ventilation is an important measure for reducing the risk of airborne infections in schools.  The most important effect is to reduce the time of residence of pathogens in the classrooms | 1. Di Gilio, A., Palmisani, J., Pulimeno, M., Cerino, F., Cacace, M., Miani, A., & de Gennaro, G. (2021). CO_2_ concentration monitoring inside educational buildings as a strategic tool to reduce the risk of Sars-CoV-2 airborne transmission. *Environmental research*, *202*, 111560. <https://doi.org/10.1016/j.envres.2021.111560> 2. Buonanno, G., Ricolfi, L., Morawska, L., & Stabile, L. (2022). Increasing ventilation reduces SARS-CoV-2 airborne transmission in schools: A retrospective cohort study in Italy's Marche region. *Frontiers in public health*, *10*, 1087087. <https://doi.org/10.3389/fpubh.2022.1087087> 3. Duill, F. F., Schulz, F., Jain, A., Krieger, L., van Wachem, B., & Beyrau, F. (2021). The Impact of Large Mobile Air Purifiers on Aerosol Concentration in Classrooms and the Reduction of Airborne Transmission of SARS-CoV-2. *International journal of environmental research and public health*, *18*(21), 11523. <https://doi.org/10.3390/ijerph182111523> 4. Villanueva, F., Notario, A., Cabañas, B., Martín, P., Salgado, S., & Gabriel, M. F. (2021). Assessment of CO_2_ and aerosol (PM_2.5_, PM_10_, UFP) concentrations during the reopening of schools in the COVID-19 pandemic: The case of a metropolitan area in Central-Southern Spain. *Environmental research*, *197*, 111092. <https://doi.org/10.1016/j.envres.2021.111092> 5. Curtius, J., Granzin, M., & Schrod, J. (2021). Testing mobile air purifiers in a school classroom: Reducing the airborne transmission risk for SARS-CoV-2. *Aerosol Science and Technology*, *55*(5), 586–599. <https://doi.org/10.1080/02786826.2021.1877257> 6. Vouriot, C. V. M., Burridge, H. C., Noakes, C. J., & Linden, P. F. (2021). Seasonal variation in airborne infection risk in schools due to changes in ventilation inferred from monitored carbon dioxide. *Indoor air*, *31*(4), 1154–1163. <https://doi.org/10.1111/ina.12818> 7. Schibuola, L., & Tambani, C. (2021). High energy efficiency ventilation to limit COVID-19 contagion in school environments. *Energy and buildings*, *240*, 110882. <https://doi.org/10.1016/j.enbuild.2021.110882> 8. Pavilonis, B., Ierardi, A. M., Levine, L., Mirer, F., & Kelvin, E. A. (2021). Estimating aerosol transmission risk of SARS-CoV-2 in New York City public schools during reopening. *Environmental research*, *195*, 110805. <https://doi.org/10.1016/j.envres.2021.110805> 9. Park, S., Choi, Y., Song, D., & Kim, E. K. (2021). Natural ventilation strategy and related issues to prevent coronavirus disease 2019 (COVID-19) airborne transmission in a school building. *The Science of the total environment*, *789*, 147764. https://doi.org/10.1016/j.scitotenv.2021.147764 10. Zivelonghi, A., & Lai, M. (2021). Mitigating aerosol infection risk in school buildings: The role of natural ventilation, volume, occupancy and CO2 monitoring. *Building and Environment*, *204*, 108139. <https://doi.org/10.1016/j.buildenv.2021.108139> 11. XU, Y., CAI, J., LI, S., HE, Q., & ZHU, S. (2021). Airborne infection risks of SARS-CoV-2 in U.S. schools and impacts of different intervention strategies. *Sustainable Cities and Society*, *74*, 103188. <https://doi.org/10.1016/j.scs.2021.103188> 12. Shen, J., Kong, M., Dong, B., Birnkrant, M. J., & Zhang, J. (2021). A systematic approach to estimating the effectiveness of multi-scale IAQ strategies for reducing the risk of airborne infection of SARS-CoV-2. *Building and environment*, *200*, 107926. <https://doi.org/10.1016/j.buildenv.2021.107926> 13. Dai, H., & Zhao, B. (2020). Association of the infection probability of COVID-19 with ventilation rates in confined spaces. *Building simulation*, *13*(6), 1321–1327. <https://doi.org/10.1007/s12273-020-0703-5> 14. Stabile, L., Pacitto, A., Mikszewski, A., Morawska, L., & Buonanno, G. (2021). Ventilation procedures to minimize the airborne transmission of viruses in classrooms. *Building and environment*, *202*, 108042. https://doi.org/10.1016/j.buildenv.2021.108042 |
| A multi-layered strategy for COVID-19 infection prophylaxis in schools: A review of the evidence for masks, distancing, and ventilation | Robert S. McLeod, Christina J. Hopfe, Eberhard Bodenschatz,  Heinz-Jörn  Moriske, Ulrich Pöschl, Tunga Salthammer, Joachim Curtius,  Frank Helleis, Jennifer Niessner, Caroline Herr, Thomas Klimach,  Martin Seipp, Thomas Steffens, Christian Witt, Stefan N. Willich | 2022 | Review | Up to June 12 2022 | Schools | High-quality  masks and regular  testing are the most effective measures,  natural and mechanical ventilation reduce infection risks in classrooms by over 80%. Ventilation systems and air purifiers cannot obviate the need  for masking and physical distancing | 1. Ferrari, S., Blázquez, T., Cardelli, R., Puglisi, G., Suárez, R., & Mazzarella, L. (2022). Ventilation strategies to reduce airborne transmission of viruses in classrooms: A systematic review of scientific literature. *Building and environment*, *222*, 109366. <https://doi.org/10.1016/j.buildenv.2022.109366> 2. Birmili, W., Selinka, H. C., Moriske, H. J., Daniels, A., & Straff, W. (2021). Lüftungskonzepte in Schulen zur Prävention einer Übertragung hochinfektiöser Viren (SARS-CoV‑2) über Aerosole in der Raumluft [Ventilation concepts in schools for the prevention of transmission of highly infectious viruses (SARS-CoV-2) by aerosols in indoor air]. *Bundesgesundheitsblatt, Gesundheitsforschung, Gesundheitsschutz*, *64*(12), 1570–1580. https://doi.org/10.1007/s00103-021-03452-4 . 3. More infections in poorly ventilated classrooms (EMPA study https://www.sciena.ch/it/research/more-infections-in-poorly-ventilated-classrooms.html). 4. Buonanno, G., Ricolfi, L., Morawska, L., & Stabile, L. (2022). Increasing ventilation reduces SARS-CoV-2 airborne transmission in schools: A retrospective cohort study in Italy's Marche region. *Frontiers in public health*, *10*, 1087087. <https://doi.org/10.3389/fpubh.2022.1087087> 5. Schibuola, L., & Tambani, C. (2021). High energy efficiency ventilation to limit COVID-19 contagion in school environments. *Energy and buildings*, *240*, 110882. https://doi.org/10.1016/j.enbuild.2021.110882 . 6. Park, S., Choi, Y., Song, D., & Kim, E. K. (2021). Natural ventilation strategy and related issues to prevent coronavirus disease 2019 (COVID-19) airborne transmission in a school building. *The Science of the total environment*, *789*, 147764. <https://doi.org/10.1016/j.scitotenv.2021.147764> 7. Stabile, L., Pacitto, A., Mikszewski, A., Morawska, L., & Buonanno, G. (2021). Ventilation procedures to minimize the airborne transmission of viruses in classrooms. *Building and environment*, *202*, 108042. <https://doi.org/10.1016/j.buildenv.2021.108042> 8. Curtius, J., Granzin, M., & Schrod, J. (2021). Testing mobile air purifiers in a school classroom: Reducing the airborne transmission risk for SARS-CoV-2. *Aerosol Science and Technology*, *55*(5), 586–599. <https://doi.org/10.1080/02786826.2021.1877257> 9. Uhde, E., Salthammer, T., Wientzek, S., Springorum, A., & Schulz, J. (2022). Effectiveness of air-purifying devices and measures to reduce the exposure to bioaerosols in school classrooms. *Indoor air*, *32*(8), e13087. https://doi.org/10.1111/ina.13087 . |
| Airborne transmission of SARS-CoV-2 in indoor environments: A comprehensive review | Jialei Shen, Meng Kong, Bing Dong, Michael J. Birnkrant & Jianshun Zhang | 2021 | Comprehensive review |  | Schools | Mobile air purifiers reduce the transmission risk of COVID-9 | 1. Curtius, J., Granzin, M., & Schrod, J. (2021). Testing mobile air purifiers in a school classroom: Reducing the airborne transmission risk for SARS-CoV-2. *Aerosol Science and Technology*, *55*(5), 586–599. https://doi.org/10.1080/02786826.2021.1877257 |
| A Systematic Review on Indoor Environmental Quality in Naturally Ventilated School Classrooms: A Way Forward | Nishant Raj Kapoor , Ashok Kumar , Chandan Swaroop Meena ,  Anuj Kumar , Tabish Alam ,Nagesh Babu Balam, and Aritra Ghosh | 2021 | Systematic review |  |  | No proper evidence to  prevent the transmission of COVID-19 inside  naturally ventilated school. Best way: fresh air after proper  filtration and mechanical ventilation |  |
